# Rapid Stiffness Mapping in Soft Biologic Tissues With Micrometer Resolution Using Optical Multifrequency Time‐Harmonic Elastography

**DOI:** 10.1002/advs.202410473

**Published:** 2024-12-16

**Authors:** Jakob Jordan, Noah Jaitner, Tom Meyer, Luca Bramè, Mnar Ghrayeb, Julia Köppke, Oliver Böhm, Stefan Klemmer Chandia, Vasily Zaburdaev, Liraz Chai, Heiko Tzschätzsch, Joaquin Mura, Jürgen Braun, Anja I.H. Hagemann, Ingolf Sack

**Affiliations:** ^1^ Department of Radiology Charité – Universitätsmedizin Berlin 10117 Berlin Germany; ^2^ Department of Hematology/Oncology Charité – Universitätsmedizin Berlin 10117 Berlin Germany; ^3^ German Cancer Consortium (DKTK)—German Cancer Research Center (DKFZ) 69120 Heidelberg Germany; ^4^ The Center for Nanoscience and Nanotechnology Edmond J. Safra Campus The Hebrew University of Jerusalem Jerusalem 91901 Israel; ^5^ Institute of Chemistry Edmond J. Safra Campus The Hebrew University of Jerusalem Jerusalem 91901 Israel; ^6^ Department of Biology Friedrich‐Alexander‐Universität Erlangen‐Nürnberg 91058 Erlangen Germany; ^7^ Max‐Planck‐Zentrum für Physik und Medizin 91054 Erlangen Germany; ^8^ Institute of Medical Informatics Charité – Universitätsmedizin Berlin 10117 Berlin Germany; ^9^ Department of Mechanical Engineering Universidad Técnica Federico Santa María Santiago 8330015 Chile

**Keywords:** biofilms, multifrequency shear waves, optical microscopy, soft tissue stiffness, time harmonic elastography elastography, zebrafish

## Abstract

Rapid mapping of the mechanical properties of soft biological tissues from light microscopy to macroscopic imaging can transform fundamental biophysical research by providing clinical biomarkers to complement in vivo elastography. This work introduces superfast optical multifrequency time‐harmonic elastography (OMTHE) to remotely encode surface and subsurface shear wave fields for generating maps of tissue stiffness with unprecedented detail resolution. OMTHE rigorously exploits the space‐time propagation characteristics of multifrequency time‐harmonic waves to address current limitations of biomechanical imaging and elastography. Key solutions are presented for stimulation, wave decoding, and stiffness reconstruction of shear waves at multiple harmonic frequencies, all tuned to provide consistent stiffness values across resolutions from microns to millimeters. OMTHE's versatility is demonstrated by simulations, phantoms, *Bacillus subtilis* biofilms, zebrafish embryos and adult zebrafish, reflecting the diversity of biological systems from a mechanics perspective. By zooming in on stiffness details from coarse to finer scales, OMTHE has the potential to advance mechanobiology and offers a way to perform biomechanics‐based tissue histology that consistently matches in vivo time‐harmonic elastography in patients.

## Graphical abstract

This study introduces an optical method for rapid stiffness mapping with micrometer resolution using time‐harmonic shear waves at multiple excitation frequencies. The new method facilitates direct comparison with in vivo elastography for diagnostic purposes and presents maps with unprecedented levels of detail in a variety of biomedical applications, from zebrafish to biofilms.

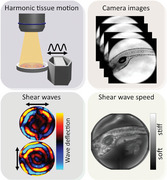

## 1 Introduction

The shear modulus of biological tissue is a fundamental parameter that characterizes stiffness and holds the key to a plethora of insights into biophysical structures and parenchymal functions. For example, stiffness is a guiding principle for mechanosensing, which in turn enables cells to act collectively in both normal and abnormal conditions as diverse as embryonic development,^[^ ^1^, ^2^, ^3^ ^]^ tumor progression,^[^ ^4^, ^5^ ^]^ or microbial pattern formation.^[^ ^6^, ^7^ ^]^ Mechanical cues allow cells to interact with their environment across the entire phylogenetic tree.^[^ ^8^, ^9^ ^]^ In medical imaging, stiffness measurement by magnetic resonance elastography (MRE) or ultrasound based elastography (USE) has been established as a diagnostic marker in a range of diseases from fibrosis to cancer.^[^ ^10^ ^]^

To explore the wealth of information that is conveyed by soft‐tissue mechanical properties, investigators have developed a number of methods including high‐throughput single‐cell deformability tests^[^ ^11^ ^]^ and optical stretching,^[^ ^12^ ^]^ surface‐based methods such as atomic force microscopy (AFM)^[^ ^13^ ^]^ and shear rheometry,^[^ ^14^ ^]^ nondestructive techniques such as Brillouin microscopy,^[^ ^15^ ^]^ optical coherence elastography (OCE)^[^ ^16^, ^17^, ^18^ ^]^ and shear wave‐based MRE and USE for the investigation of microsamples^[^ ^19^ ^]^ to full organs in patients.^[^ ^20^ ^]^

Despite this impressive arsenal of tools, there remains a large gap between values obtained at the organ level in vivo and microtissues, hindering the translation of mechanobiology into clinical diagnostics. Therefore, we propose here optical multifrequency time‐harmonic elastography (OMTHE), which combines the versatility of optical motion detection with inverse problem solutions developed over the years in multifrequency MRE.^[^ ^21^ ^]^ On the one hand, optical methods have proven to be useful for rapid microelastography in transparent tissues or on surfaces of opaque materials^[^ ^22^, ^23^, ^24^, ^25^, ^26^, ^27^, ^28^ ^]^ (see also supplementary note 1: optical elastography methods). On the other hand, the detection of multifrequency time‐harmonic waves by light would allow to bridge length scales from micro‐resolutions to entire organs by leveraging the scaling behavior of wavelengths excited at a precisely known frequency.^[^ ^29^ ^]^ However, the high optical resolution of light cameras has never been fully exploited for stabilizing inverse problem solutions by repeated experiments at different excitation frequencies.^[^ ^22^ ^]^

OMTHE rigorously exploits the time‐space propagation characteristics of multifrequency shear waves by rapid stroboscopic sampling, novel harmonic motion detection, and multifrequency‐multicomponent wave inversion. This allows OMTHE to map stiffness in fractions of a second with µm resolution and values that are directly comparable to established MRE measurements. The versatility of OMTHE is demonstrated in tissue systems as diverse as biofilms of different ages and both zebrafish embryos and adults. *Bacillus subtilis* biofilms are prokaryotic analogues of tissue‐like structures^[^ ^30^ ^]^ exhibiting cell differentiation, heterogeneous phenotype distribution, extracellular matrix production, and formation of intricate vasculature‐like water‐filled channels for nutrient distribution.^[^ ^31^ ^]^ Zebrafish embryos, whose genes are ≈70% orthologous with humans,^[^ ^32^ ^]^ provide a prototypical and versatile model for studying tissue differentiation and full organ formation.^[^ ^33^, ^34^ ^]^

There is currently no single method that can simultaneously measure stiffness in such diverse systems: biofilms are opaque and grow primarily in two dimensions while zebrafish embryos are transparent and develop complex 3D organs. As will be shown, OMTHE allows us to study these systems using exactly the same principles of mechanical stimulation and wave analysis, providing, for the first time, maps that can be compared across tissues and species at different length scales and resolutions.

## 2 Results

### 2.1 Performance of OMTHE

**Figure 1:**
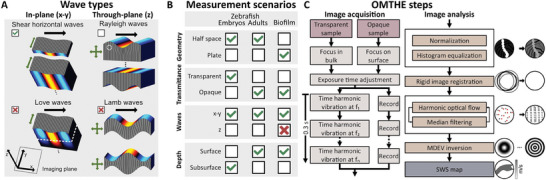
Principles of surface waves harnessed by OMTHE, studied systems, and processing pipeline. A) Wave types propagating in half‐spaces and plates, including their polarization directions (green arrows) and propagation direction (black arrows) in incompressible media. Wave deflections are shown on a blue to yellow color scale (positive to negative amplitudes). The orientation of the image plane relative to the coordinate axes is shown in the lower left corner. The preferred wave scenario in OMTHE is the SH wave, which probes *SWS* related to intrinsic shear modulus of the tissue independent of the layer thickness (green checkmark). Unfavorable scenarios are Rayleigh, Lamb, and Love waves (red crosses: biased *SWS*, open checkbox: unbiased *SWS* possible). While Rayleigh waves (mainly z‐surface deflections) in incompressible and semi‐infinite media reflect the subsurface shear modulus *G*′,^[^ ^37^ ^]^ z‐bending deflections in plates are Lamb waves, which have symmetric or antisymmetric modes whose *SWS* is influenced by a mixture of frequency, geometry, and bending modulus of the material.^[^ ^38^ ^]^ To avoid geometric bias, z‐wave measurement is not recommended in OMTHE of thin films or tissue slices. Similarly, Love waves (xy‐deflections in layer media) should be avoided due to a possible geometric bias introduced by elastic deformations of both top layer and support.^[^ ^39^ ^]^ However, as discussed in the Appendix, Love waves are unlikely in OMTHE unless multiple wavelengths fit into the layer thickness and the substrate is elastically deformed by the surface waves. B) Overview of different measurement scenarios to accommodate different sample characteristics. Scenarios used in this work are indicated by a green checkmark while a red cross indicates biased *SWS* due to Lamb waves in biofilms. C) Overview of basic OMTHE measurement and analysis steps. Depending on the type of sample, optical lenses are focused in the bulk or on the tissue surface. The exposure time is adjusted according to the lighting conditions. Next, the vibration is triggered, and the images are captured and stored in the camera. After transfer to a workstation, the images are normalized, and histogram equalization is applied to correct for inhomogeneous intensity. The images are then registered to the first image of a series to remove jitter and compression waves. Next, harmonic optical flow (HOF) is used to decode multifrequency xy‐shear waves, which are then converted to *SWS* maps by multifrequency dual elasto‐visco (MDEV) inversion. For more details see the Methods section.

We first reviewed the basic assumptions in material properties, geometries, and optical properties underlying OMTHE. Acquisition of shear waves by remote optical cameras required excitation units (actuators) for inducing xy‐polarized waves with respect to the xy‐imaging plane. **Figure** **1** summarizes the principal wave components and optical measurement scenarios occurring in incompressible soft biological tissues whose geometries can be considered semi‐infinite or layered. The imaging plane was adjusted by focusing the optical system either on the surface in opaque tissues or on the subsurface in transparent tissues. The preferred OMTHE wave scenario was that of shear horizontal (SH) waves which are characterized by polarization and propagation directions within the xy‐imaging plane and a shear wave speed $SWS=\sqrt{G^{\prime }/\rho }$ that is governed by the material's storage modulus $\;G^{\prime }$ assuming purely elastic material properties. Note that, ρ = 1 kg L^−1^ unit density is a common assumption in elastography of incompressible media.^[^ ^35^, ^36^ ^]^ Due to their dependence on the object geometry, z‐deflections as well as Love waves (xy‐waves in special layered scenarios, see discussion in the appendix) were considered unfavorable for OMTHE (Figure 1A). xy‐shear waves encoded by in‐plane motion of particles and optical features were detected in either reflection or transmission mode of our optical systems, depending on the geometry and optical properties of the investigated tissue, as summarized in Figure 1B. Figure 1C outlines basic OMTHE acquisition and postprocessing steps.

**Figure 2:**
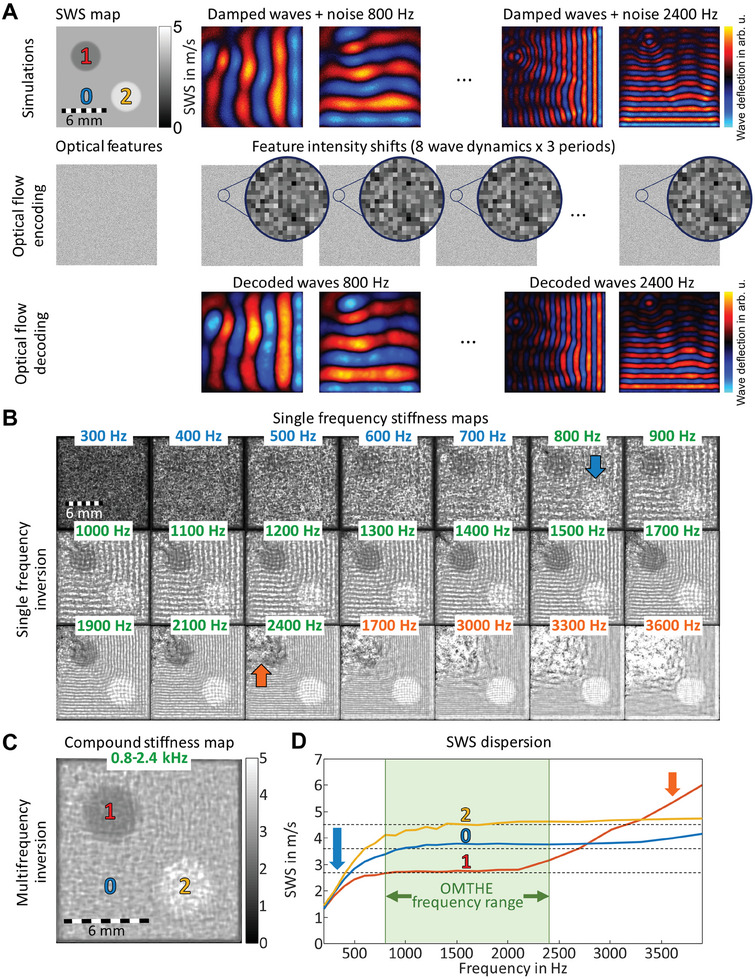
Simulated OMTHE performance. A) First row: ground‐truth *SWS* map with two inclusions (*SWS* = 2.7, 3.6, 4.5 m s^−1^ for inclusion 1, background 0 and inclusion 2, respectively) as well as simulated shear waves (x‐ and y‐components) for 800 and 2400 Hz frequencies with added noise. Second row: simulated camera image with random scatterers that encode the harmonic particle motion within the imaging plane which is in the order of 10% of pixel size and, thus, visually apparent only as animation provided in Movie S1 (Supporting Information). Nevertheless, HOF correctly decoded the wavefields as shown for 800 and 2400 Hz despite added Gaussian noise of 10%. B) Single frequency inversion results. At low frequencies, noise dominates longer wavelengths while at high frequencies the inversion fails in soft areas due to enhanced wave damping. C) Multifrequency inversion results. Compound *SWS* map based on all frequencies between 800 and 2400 Hz show the correct location and stiffness of both inclusions. D) *SWS* values for inclusions 1 and 2 and background 0 as well as ground truth (horizontal dashed lines). Good agreement is obtained when averaging *SWS* values in the OMTHE frequency range between 800 and 2400 Hz (*SWS* = 2.7, 3.5, 4.3 m s^−1^ for 1, 0, 2) while underestimation (blue arrow) and overestimation (red arrow) are expected due to noisy features^[^ ^42^ ^]^ and evanescent phase artifacts,^[^ ^43^ ^]^ respectively.

Multifrequency wave excitation and acquisition is a key element of OMTHE. How multiple frequencies improve the consistency of stiffness maps is demonstrated in **Figure** **2** based on finite difference wave simulations. Here, and in the following, we used *SWS* as a proxy of shear modulus (*SWS* ^2^ ∝ *G*′) obtained from complex‐valued xy‐wave field components ${\hat{u}}_{j}\left(x,y,f\right)$ as detailed in the Appendix. Due to the small field‐of‐view (FoV) ranging from 2 to 18 mm, higher mechanical excitation frequencies between 800 and 2400 Hz were used similar to the frequency range of preclinical multifrequency MRE.^[^ ^40^ ^]^ Figure 2A demonstrates the sensitivity of novel harmonic optical flow (HOF) decoding presented in the Appendix for the retrieval of xy‐shear waves encoded in random noise patterns. Although no deflection was visually apparent (see Movie S1, Supporting Information), HOF‐decoded waves appeared very similar to ground truth waves. Nevertheless, single frequency direct inversion yielded artifacts and wave residues in *SWS* maps due to overlapping effects of noise, wave damping and non‐harmonic waveforms. However, as shown in Figure 2C, the consistency of frequency‐compound *SWS* maps generated by averaging magnitude wave images and magnitude Laplacian images over the full OMTHE frequency range *prior* to inversion^[^ ^41^, ^42^ ^]^ was greatly improved in terms of both background variations and boundary fidelity. *SWS* values over frequency, shown in Figure 2D, further motivated multifrequency inversion in OMTHE from 800 to 2400 Hz frequency. Within this range, inclusions and background *SWS* values were relatively stable with expected underestimations at low wavenumbers (<800 Hz) and overestimations at higher wavenumbers (>2400 Hz) due to noisy features^[^ ^42^ ^]^ and evanescent phase artifacts,^[^ ^43^ ^]^ respectively.^[^ ^44^ ^]^

### 2.2 Phantoms

**Figure 3:**
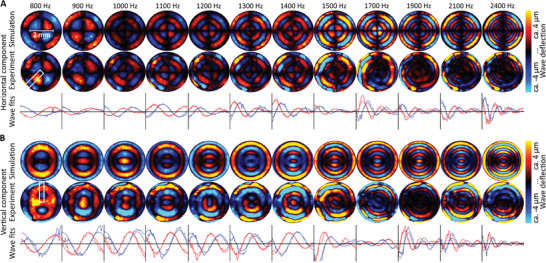
OMTHE xy‐wave components based on experimental and simulated displacements in a homogeneous cylindrical phantom vibrated along the vertical (y‐) axis. Horizontal (x‐) A) and vertical (y‐) B) wavefield components across all frequencies. Simulated wavefields are shown in the first rows and measured wavefields are shown below. Radial profiles of the real (red) and imaginary parts (blue) of the waves, as used for the analysis in Figure 4A, are shown below the wavefields. Maximum wave deflection was on the order of 4 µm, as measured by laser distance measurement (see Methods). Line profiles were drawn inside the white rectangles.

**Figure 4:**
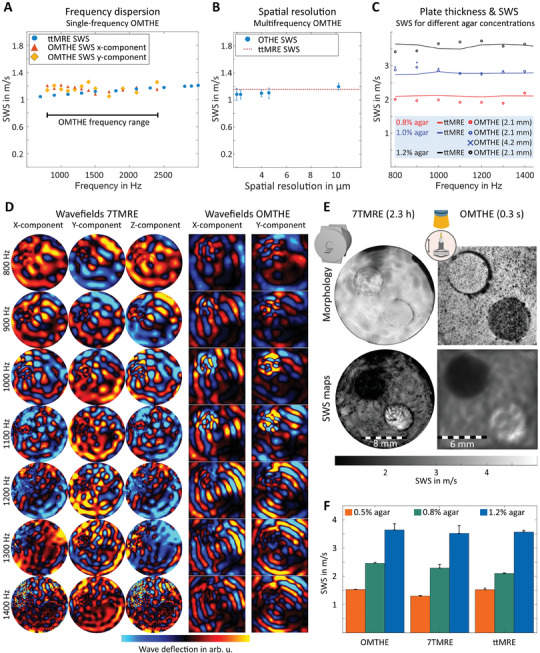
OMTHE in agar and US gel phantoms. A) Frequency‐resolved *SWS* obtained from profile fits as shown in Figure 3. B) *SWS* obtained from MDEV inversion applied to OMTHE and ttMRE wave images, matching to the ground truth across spatial resolutions. C) Frequency‐resolved OMTHE and ttMRE at three agar concentrations and agar layer thicknesses showing good agreement between measurement methods without thickness effect on *SWS*. D) Snapshots of all wavefields used for both MRE and OMTHE in a heterogenous agar phantom with two inclusions of different stiffnesses. E) A T2‐weighted MRE magnitude image and camera image shown the locations of the inclusions in the first row, with *SWS* maps from both methods shown in the second row. The measurement time for 7TMRE was 2.3 h while OMTHE took 0.3 s. F) The resulting mean *SWS* with standard deviation based on four repeated measurements, with good agreement between the methods.

We experimentally tested the consistency of OMTHE in phantoms using different excitation frequencies, spatial resolutions, phantom material stiffnesses and geometries. **Figure** **3** shows in‐plane xy‐wave fields in a cylindrical phantom with a pixel resolution of 4 µm × 4 µm for each frequency. The apparent similarity of experimental and simulated wavefields illustrates the overall consistency of our approach. Quantitative agreement between OMTHE and simulations is demonstrated by line profiles and fits of wavelengths providing *SWS* values for all frequencies. In addition, **Figure** **4**A shows that OMTHE agrees with tabletop MRE (ttMRE) across all frequencies with a mean deviation of less than 3%. Combining all frequencies to compound *SWS* maps provided consistent values in the range of the ground truth obtained by ttMRE with deviations less than 3% (Figure 4B). Stable *SWS* solutions were also obtained using various lens systems with resolutions ranging from 2 µm ×  2 µm to 10 µm × 10 µm, suitable for encoding xy‐waves with negligible inversion bias^[^ ^44^ ^]^ in our preferred frequency range. We further tested the validity of OMTHE's main assumption that xy‐waves reflect shear waves in a plain‐strain scenario where *SWS* can be considered as a surrogate of shear modulus independent of layer thickness. Figure 4C shows frequency resolved OMTHE in agar phantoms of variable thickness and stiffness in comparison to MRE. No significant difference was found between both modalities with overall less than 11% deviations (P = 0.28). Figure 4F,D show experimental wave images and *SWS* maps of agar phantoms with inclusions obtained by both MRE at 7 Tesla (7TMRE) and OMTHE. Figure 4E shows that *SWS* values recovered from the different phantom areas were very similar in OMTHE and 7TMRE without statistically significant difference (P = 0.17). Having validated the OMTHE setup under controlled conditions, it was applied to various examples of biological tissues.

### 2.3 Zebrafish

**Figure 5:**
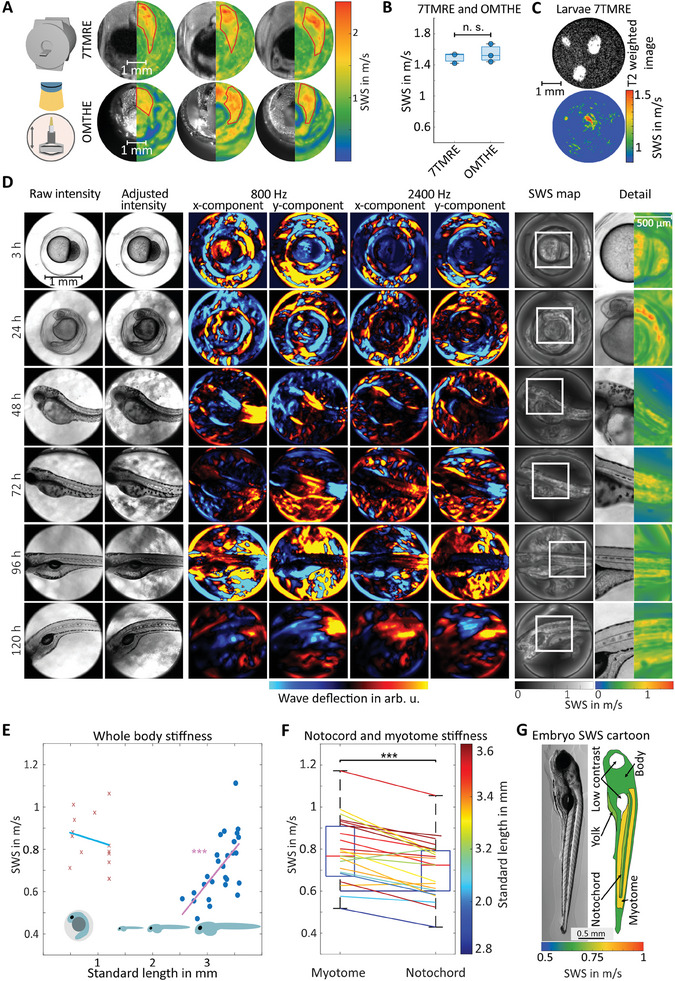
OMTHE in zebrafish. A) Three adult zebrafish, sectioned transversely after 7TMRE and examined by OMTHE. The left half shows T2 weighted images (top row) and the camera image (bottom row), while the right half shows *SWS* maps. The muscle region is outlined in red. B) Comparison of mean *SWS* obtained by 7TMRE and OMTHE; differences are not significant (P = 0.50). C) 7TMRE of three zebrafish embryos, with T2‐weighted MRE magnitude image at top and *SWS* map at bottom. D) From left to right: raw intensity images as captured by the high‐speed camera, normalized camera images, waveforms for the lowest and highest frequency measured in both x‐ and y‐directions, *SWS* map, and magnified region of interest for one sample per age group. E) Mean *SWS* of zebrafish embryos at different standard lengths. After hatching, *SWS* of the embryos increases linearly at a rate of 0.3300 ± 0.0003 ms mm^−1^ over the studied age range. F) *SWS* differs between zebrafish anatomical regions, here larval notochord and myotome after hatching (*p* < 0.001). G) Cartoon of examined regions of interest in hatched zebrafish embryos with color coded mean *SWS*. Areas not resolvable by OMTHE are shown in white. For a complete set of wave images of one zebrafish, see Figure S1 (Supporting Information), for an overview of embryo development, see Figure S2 (Supporting Information). *p < 0.05. **p < 0.01, ***p < 0.001, n.s. not significant.

The zebrafish model^[^ ^45^, ^46^ ^]^ has greatly facilitated biomedical research due to its rapid development, large clutch size, high number of human‐orthologous genes, and ease of husbandry. While zebrafish are well understood on the genetic, behavioral, and anatomic levels,^[^ ^32^, ^34^ ^]^ biomechanical investigations of whole fish at any stage are challenging due to their small size and heterogeneity. We recently established high‐resolution 7TMRE in the adult zebrafish^[^ ^19^ ^]^ and used this method here as a reference for OMTHE. *SWS* measured in the trunk muscle agreed between both modalities with 1.5 ± 0.2 m s^−1^ for OMTHE and 1.5 ± 0.1 m s^−1^ for 7TMRE (P = 0.50), with a maximum difference of only 0.1 m s^−1^ (see **Figure** **5**A,B). However, 7TMRE provided a spatial resolution of only 60 µm, which did not allow investigation of zebrafish embryos (see Figure 5C).

Figure 5D shows camera images, wavefields of lowest and highest frequencies, and *SWS* maps obtained in zebrafish embryos at different ages. The wave images showed favorable characteristics for multifrequency direct inversion as they appear noise‐free with a high SNR of 80 ± 5 dB due to the excellent fidelity of the optical lenses and intrinsic noise‐suppression in HOF. Therefore, direct inversion could be used without prior filtering, which enabled high‐resolution *SWS* mapping. In all fish, *SWS* was higher in myotome than in notochord tissue (0.8 ± 0.1 m s^−1^ vs. 0.7 ± 0.1 m s^−1^, *P* = 6.1 × 10^−5^) but lower than obtained in muscles of adult zebrafish (2.9 ± 0.5 m s^−1^). This difference could be attributable to developmental changes as suggested by the results presented in Figure 5E, where the increase in *SWS* in hatched fish over increasing standard length (SL)^[^ ^47^ ^]^ is plotted. We found a linear *SWS* increase of 0.3300 ± 0.0003 m s^−1^ (*R* = 0.65, *P* = 2.5 × 10^−4^) per 1 mm standard length, which results in >1.5 m s^−1^ for 5 mm SL and explains the observed difference between freshly hatched fish and adult fish. In contrast to the development of stiffness in hatched fish, we did not observe *SWS* changes in either prehatched tissue (0.8 ± 0.1 m s^−1^, R = −0.22, *P* = 0.47) or yolk (0.7 ± 0.1 m s^−1^, R = −0.09, *P* = 0.59) over SL.

### 2.4 Biofilms

Biofilms are industrially and clinically relevant colonies of prokaryotes embedded in self‐secreted polymeric extracellular matrix (ECM).^[^ ^48^ ^]^ Characteristic features of biofilms are their ability to colonize virtually any interface and their resistance to environmental stressors, including mechanical forces and the effects of antimicrobial agents.^[^ ^49^, ^50^ ^]^ Biofilms are increasingly considered analogues or sometimes even parts of human tissue.^[^ ^51^ ^]^ The mechanical interplay of biofilms and their substrate^[^ ^52^ ^]^ can lead to formation of a network of wrinkles that cover water‐filled channels used for nutrient transport.^[^ ^52^, ^53^ ^]^

**Figure 6:**
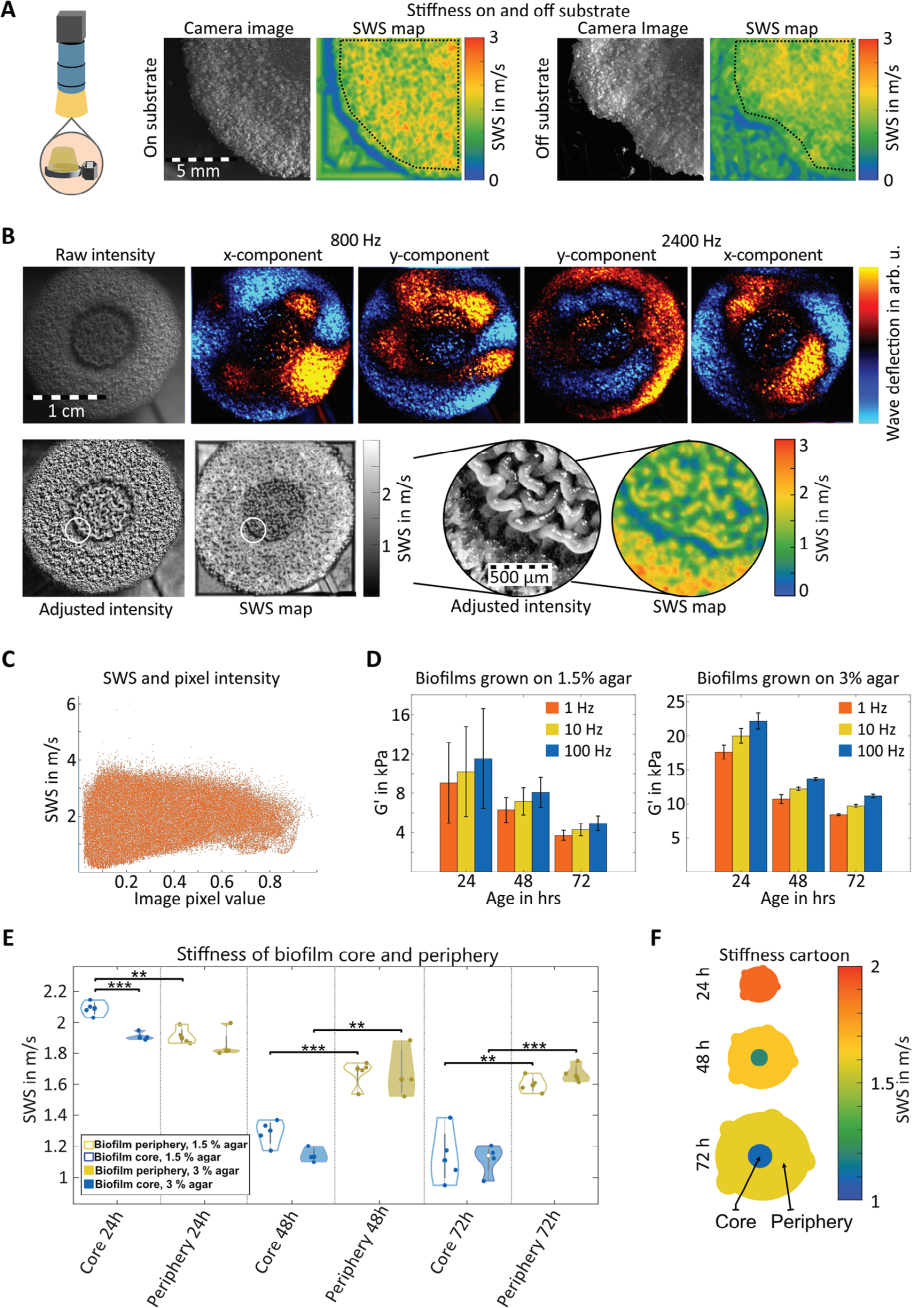
OMTHE in biofilms. A) Quadrant of a Bacillus subtilis biofilm grown on 1.5% agar for 48 h. Left pair: biofilm measured on agar surface with *SWS* map on the left and camera image on the right. Right pair: the same biofilm removed from the agar and remeasured on a plastic dish. Mean *SWS* values for the quadrant are the same on and off the agar. B) Example data from one biofilm grown on 3% agar after 48 h. First row from left to right: raw intensity images as captured by the high‐speed camera and wave images for the lowest and highest frequency measured in both x and y directions. Second row: adjusted camera images, *SWS* map, central area of the biofilm magnified, with the adjusted camera image and *SWS* map of the area shown. C) Scatter plot of adjusted image intensity and corresponding *SWS* per pixel for all biofilms. D) Rheology measurements for biofilms grown on both agar conditions, with storage modulus G′ for each measured age and frequency shown. Both conditions show a significant decrease in G′ with biofilm age (P = 7.5 × 10^−5^ for 1.5% agar substrate and *P* = 6.8 × 10^−4^ for 3% agar substrate). Error bars represent standard deviation between repetitions. E) Comparison of biofilm *SWS* in the core and periphery for biofilm grown on 1.5% agar (blue) and 3% agar (red). F) Cartoon of stiffness progression with age in both regions. For a complete set of wave images of one biofilm, see Figure S1 (Supporting Information). **p < 0.01, ***p < 0.001.

To study the interplay between local mechanical properties and biofilm development, we used OMTHE for mapping the material properties of *Bacillus subtilis* as a biofilm‐forming model. We first tested whether xy‐waves in the plane‐strain scenario provided consistent *SWS* values in biofilms without being confounded by the adhesion strength to and stiffness of their substrate. **Figure** **6**A shows biofilm *SWS* maps from the same sample, both grown on the substrate and after being removed from the substrate and measured on the bottom of a plastic dish. In both scenarios, the same vibrations were induced. Encouragingly, values were the same for the no‐substrate (1.5 ± 0.3 m s^−1^) and on‐substrate case (1.5 ± 0.3 m s^−1^), suggesting that our plane strain xy‐waves probed intrinsic biofilm properties and were unaffected by adhesion to the underlying support. However, substrates on which a biofilm develops can affect the biofilm's material properties and growth.^[^ ^53^ ^]^ To test whether substrate stiffness affects OMTHE *SWS* values, biofilms grown on substrates of different stiffness (1.5% and 3% agar content) were studied at three time points of 24, 48, and 72 h post seeding (Figure 6B,D). Wave images presented in Figure 6B display pronounced features due to wave diffraction on the scale of tissue heterogeneities visible in light micrographs. Diffraction revealed motile structures and abundant slip interfaces within the biofilms as a corollary of structure and composition. Mean *SWS* values shown in Figure 6E quantified the mechanical distinction between central and peripheral regions of the biofilms, with Figure 6F visualizing the same trend. Notably, *SWS* appeared to be unaffected by optical properties, as suggested by the bright core area in the biofilms shown in Figure 6B, while dark intensities were observed in *SWS* maps. Moreover, a correlation between *SWS* and optical pixel intensities, which pooled all biofilm data, was clearly negated in Figure 6C. The difference in *SWS* between the soft central region and the stiffer peripheral region increased consistently with age (central regions: 2.0 ± 0.3, 1.3 ± 0.3, 1.1 ± 0.3 m s^−1^, periphery: 1.9 ± 0.3, 1.7 ± 0.3, 1.6 ± 0.3 m s^−1^ at 24, 48, 72 h, with *P* = 7.4 × 10^−3^, 3.2 × 10^−4^, 1.7 × 10^−5^, respectively) showing significant softening (*R* = −0.88, *P* = 2.1 × 10^−8^), independent of substrate stiffness beyond 24 h (*P* = 0.014, otherwise *P* > 0.05). To cross‐validate these findings we performed bulk rheology measurements of biofilms of different ages and recovered the same trend (Figure 6D).

## 3 Discussion

OMTHE conceptually merges the previously separate fields of multifrequency time‐harmonic elastography and optical flow‐based stiffness mapping. The novel method enables nondestructive, high‐speed imaging of biomechanical properties from the tissue surface in opaque materials or from within the bulk of transparent tissue. Although previously described methods of light‐based elastography allow stiffness measurement at different scales, including subcellular to tissue ranges,^[^ ^22^, ^23^, ^28^, ^54^, ^55^, ^56^, ^57^, ^58^, ^59^, ^60^ ^]^ OMTHE is the only cross‐scale platform that can be easily adapted to a wide variety of scales from bacterial cell colonies to adult zebrafish. Based on the same time‐harmonic waves used in MRE, OMTHE offers the potential to bridge the gap between the histology dimension and in vivo medical imaging.

OMTHE rigorously exploits a number of key innovations from seismology^[^ ^61^ ^]^ and elastography,^[^ ^20^ ^]^ including precise excitation of harmonic motion, stroboscopic wave sampling, motion decoding by HOF, and multifrequency wave inversion optimized for the high SNR in optical images. As has been shown in phantoms and simulations, HOF decoded waves impose fewer constraints on wave inversion algorithms than other elastography techniques, including MRE, for two reasons: first, rigid motion and unwanted compression waves are cancelled out using image registration. Second, due to the excellent fidelity of optical systems, OMTHE SNR is orders of magnitude higher than that of MRE despite smaller pixel sizes. The wave SNR, as calculated using the Donoho method,^[^ ^62^ ^]^ was 80 ± 5, 58 ± 2, and 40 ± 5 dB in phantoms, zebrafish, and biofilms respectively, which is up to 10^5^ times higher than achieved in state‐of‐the‐art MRE.^[^ ^19^ ^]^ As a result, OMTHE inversion does not require filters to suppress compression waves or noise, which is a significant advantage considering current resolution limits in time‐harmonic elastography due to bandpass filters.^[^ ^44^, ^63^ ^]^ In addition to these superior noise characteristics compared to other elastography methods, OMTHE can overcome challenging optical conditions by combining multiple images with different exposure times using high dynamic range algorithms. Tissue heterogeneities can also be better addressed by OMTHE inversion due to the 10 to 100 times higher pixel‐to‐wavelength ratio compared to MRE.^[^ ^44^ ^]^

Such a multiscale optical imaging technique, which provides µm resolution within less than a second of scan time, makes new experiments possible. For example, 7TMRE has achieved pixel edge sizes of 40 µm in 600 µm thick slices with scan times of more than 16 min per frequency^[^ ^19^ ^]^ and cannot resolve zebrafish embryos as shown in Figure 5C. OCT‐based reverberant elastography achieves 20 µm lateral resolution in up to 2 mm depth within 8 min scan time for a single harmonic frequency with extensive data transfer time during which no new acquisitions can be performed. A breakthrough in time is optical‐flow based elastography with high‐speed cameras for single frequency harmonic micro elastography^[^ ^22^ ^]^ or transient elastography on surfaces,^[^ ^23^ ^]^ which, however, cannot generate pixel resolved stiffness maps. AFM achieves stiffness maps with quasi‐static nano indentation of thin tissue slices and has been established as a reference modality for micro tissue mechanical examinations including those of zebrafish.^[^ ^64^ ^]^ However, AFM maps are relatively sparse in detail because the technique relies on time‐consuming point‐based scanning procedures. Furthermore, AFM values are based on contact mechanics, which are difficult to translate into the range of *SWS* used by in vivo elastography.^[^ ^29^ ^]^

The maps of zebrafish embryos presented here resolve unprecedented detail in stiffness. This allowed us to study stiffening in myotome and notochord in the course of embryonic development. The observed correlation of whole‐body stiffness over standard length is an encouraging result as it provides a linear model to interpolate developmental changes to the specific tissue mechanical state of the fish. This could also help determine at which age xenografts can be seeded in the zebrafish model at a normalized tissue stiffness independent of animal size. Consistent with our data, microdeformation analysis has previously shown that yolk sac stiffness remains relatively constant during development.^[^ ^65^ ^]^ Brillouin microscopy has been used to assess stiffness‐related parameters in zebrafish during development and regeneration after spinal cord injury. Consistent with our observation of softer notochord than surrounding tissue, it has been speculated that softness is necessary for effective locomotion.^[^ ^66^ ^]^ These encouraging results regarding relative stiffness changes in zebrafish tissues during disease and regeneration illustrate the importance of a quantitative stiffness mapping method for biomedical research in emerging small animal disease models.

While zebrafish larvae are too small for conventional elastography, biofilms are prohibitively thin, making OMTHE the first technique for *SWS* mapping in such challenging systems.^[^ ^67^ ^]^ The biomechanical properties of biofilms depend not only on the composition of the individual bacteria but also on their ECM, cell‐matrix interactions as well as water distribution and content.^[^ ^68^, ^69^, ^70^ ^]^ Therefore, the fundamental mechanisms leading to the mechanical robustness of soft tissues can be studied in biofilms using a technique such as the one presented here. Using OMTHE, we obtained the first quantitative and detailed stiffness maps of growing biofilms on different substrate layers. The observation that biofilm stiffness was unaffected by both substrate stiffness and layer contact suggests the intrinsic constitutive nature of OMTHE parameters in these systems. Furthermore, the obtained stiffness contrast in our biofilm maps indicates fundamental differences between mechanical structures in central and peripheral areas (see supplementary note 2: On spatial resolution in biphasic fluid‐solid biofilms). Previous work has shown that biofilms use programmed cell death to form channels that facilitate the transport of water and nutrients.^[^ ^52^ ^]^ Intriguingly, nutrient flux is driven by evaporation gradients formed by a larger surface area and local curvature in the center of the biofilm compared with the outer region.^[^ ^31^ ^]^ In addition, lower cell density in the core area associated with reduced stiffness values, as recently reported by OCE,^[^ ^71^, ^72^ ^]^ may explain the soft core of biofilms revealed by the OMTHE contrast. Our stiffness values fall into a wide range of properties reported for biofilms in the literature. Bulk rheology measurement in *B. subtilis* biofilms yielded values between 56–309 Pa (0.23‐0.56 m s^−1^ *SWS*)^[^ ^67^ ^]^ and 2250 Pa (1.5 m s^−1^ *SWS*)^[^ ^73^, ^74^ ^]^ for storage modulus, which are comparable to the results we obtained by shear rheometry (see Figure 6D). AFM measured a Young's modulus of 1.97 kPa (0.81 m s^−1^ *SWS*) in biofilms covered with ethanol (to prevent AFM tip adhesion to sticky biofilm surface), which may have induced degradation.^[^ ^74^ ^]^ Ziege et al. reported higher values of Young's modulus of *Escherichia coli* biofilms on the order of 500 kPa (12.9 m s^−1^ *SWS*) without notable effects of agar substrate stiffness on biofilm stiffness in a range of agar concentrations similar to that used here.^[^ ^75^ ^]^ Youngs modulus was converted to *SWS* values using $SWS=\sqrt{E/3\rho }$.^[^ ^35^ ^]^ In this study, higher water content was associated with lower stiffness, suggesting that an increase in water in the extracellular space due to growth causes softening. Age‐related changes in water content and ECM volume fraction could also have contributed to the observed softening of the biofilms over time. Using OMTHE to identify the specific link between the aging elements and mechanical properties in biofilms would be an important step in future work.

Although this study demonstrated the basic concepts of OMTHE in a rather broad range of systems, it cannot cover its full versatility in all possible scenarios. In‐depth studies are needed to better understand the resolution limits of harmonic decoding by HOF and wave inversion by MDEV, as well as to improve parameter consistency, lighting conditions, and frequency ranges. We have explored the optimal frequency range through simulations and phantom experiments as shown in Figures 2 and 3. Encouragingly, a plateau of stable SWS values was found within a frequency range of 0.8 to 2.4 kHz across materials and resolutions. However, zooming into finer structures such as cells may require this frequency range to be adjusted to higher values as suggested by Flé et al.^[^ ^22^ ^]^ Conversely, OMTHE can cover larger fields of view by reducing the excitation frequency to values as low as 275 Hz, as demonstrated in muscle tissue. A preview of these multiscale capabilities of OMTHE is provided by a preliminary study of in vivo muscle stiffness in supplementary note 3, showing changes in muscle stiffness based on the subsurface sensitivity of Rayleigh waves. More of these studies are needed to fully understand the potential and limitations of multifrequency shear waves as a probe of tissue stiffness in optical elastography. Finally, beyond stiffness, time‐harmonic elastography is sensitive to wave attenuation and can provide viscosity‐related parameters. Although there is a large body of MRE literature on the measurement of loss modulus and damping ratio, quantification of viscosity using time‐harmonic shear waves is more complex than stiffness quantification and therefore beyond the scope of this paper. The new window into soft tissue biomechanics opened by OMTHE may lead to new research questions that require optimized and specialized techniques of light‐based time‐harmonic elastography. As such, the method presented here is a platform for further development rather than a one‐fits‐all solution.

In summary, OMTHE is a versatile time‐harmonic elastography platform that uses optical detection of shear waves on surfaces or within transparent tissues for stiffness mapping in different materials and scenarios. For example, in transparent tissues, focusing a microscope on a cross section through the bulk allows optical encoding of shear waves in deeper structures, while surface properties of opaque materials can be studied by encoding in‐plane shear waves using optical lenses. OMTHE provides solutions to several persistent problems in stiffness mapping for biomedical research over multiple length scales, including high optical resolution, ultrafast scanning, robust motion estimation of periodic tissue deflections, and multifrequency inversion of harmonic wavefields without bandpass filters. OMTHE has been demonstrated to provide high‐resolution stiffness maps in zebrafish embryos during development and at surfaces of growing biofilms. These results illustrate the wide range of potential applications of OMTHE. In addition, first stiffness reference values for the frequency range between 800 and 2400 Hz are provided for zebrafish development from egg to embryo as well as for *Bacillus subtilis* biofilms from day 1 to 3 after inoculation. Taken together, fundamental developments in shear wave generation, light microscopy, optical flow detection, and wave inversion enable OMTHE to rapidly and cost‐effectively map stiffness across scales in many systems relevant to biomedical research and diagnostic medical applications.

## 4 Experimental Section

### 4.1 Phantom Preparation

**Figure 7:**
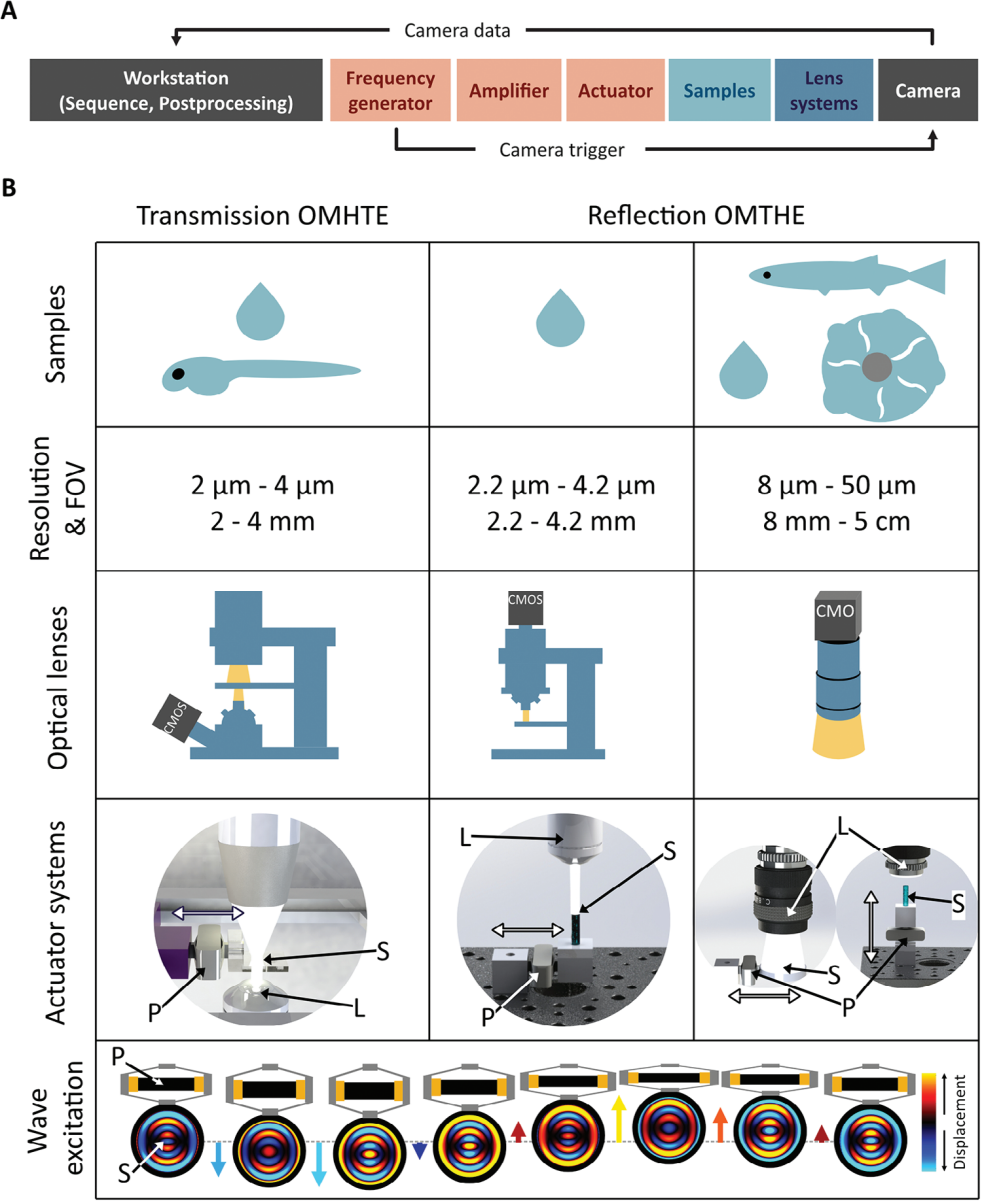
Studied materials and systems. A) Block diagram of setup and data flow: The waveform was defined in a waveform generator, which on TTL triggering sent a sinusoidal signal to an amplifier and a vibration unit. At the same time, a TTL signal synchronized to the harmonic wave triggered the image acquisition. Image magnification was defined by the lens system and the acquired images were sent back to the workstation. Lens system, actuator, and amplifier could be exchanged to accommodate different samples and resolutions. B) Schematic overview of the experiments performed in this study. Images were acquired in transmission mode (zebrafish embryo and phantom) and reflection mode (adult fish, biofilms, and phantoms) using a microscope and macro lens, respectively. 3D renderings show the different actuator setups and arrows indicate the main vibration direction. The bottom row shows how waves are excited in the sample, with displacements along the vertical axis as indicated by the colored arrows. S: sample, L: lens system, P: piezo actuator.

An ultrasound (US) gel (Medimex GmbH, Germany) phantom was used to validate OMTHE against established MRE. The US gel was mixed with scatterers (Siliziumcarbid F400, diameter 17.3 µm, Mineraliengrosshandel Hausen GmbH, Austria) to ensure good optical contrast. Additionally, homogenous agar phantoms were made by preparing 0.8%, 1.0% or 1.2% agar (Merck, Germany) water mixtures, hydrating the agar for 5 min while mixing continuously at 250 rpm and subsequently heating the mixture to 95 °C. For OMTHE measurements, 2 mL of the mixtures were then poured into 4 cm diameter petri dishes (see **Figure** **7**B), while for ttMRE measurements, 2 mL were poured into 7.5 mm inner diameter glass tubes. Measurements were performed after the agar had solidified.

The heterogeneous phantom was prepared by pouring 0.8% agar into a 16 mm wide and 25 mm deep tube with two protruding 4 mm diameter columns. After the agar solidified, the columns were removed, and the resulting voids were filled with 0.5% and 1.2% agar. Each agar‐water mixture also contained 0.2% 50 µm cellulose microcrystals (Thermo Scientific Chemicals, USA) to provide optical contrast. After the agar inserts solidified, a 3 mm thick horizontal slice of the phantom was investigated four times in different orientations using OMTHE. The remainder of the phantom was examined by 7TMRE as described below.

### 4.2 Zebrafish Preparation

Zebrafish (Danio rerio) were raised and maintained according to a standard protocol at 28 °C with a 14/10 h light‐dark cycle at the Pediatric Oncology/Hematology Department of Charité University Hospital, Berlin, Germany.^[^ ^76^ ^]^ All experiments in adult fish were performed *post mortem* as approved by the local animal ethics committee (G 0325/19, Landesamt für Gesundheit und Soziales, Berlin, Germany) and were conducted in accordance with the European Community Council Directive of November 24, 1986 (86/609/EEC). For the experiments in embryos, wild‐type fish of the Tüpfel long‐fin strain were used. All embryonic studies were performed post mortem with zebrafish embryos up to 5 days post fertilization and did not fall under the Protection of Animals Act.

Adult fish were euthanized immediately before examination by hypothermic shock as described by Wallace et al.^[^ ^77^ ^]^ For 7TMRE, three adult fish (22 months after fertilization) were scanned as described below. After 7TMRE acquisitions, the fish were sectioned transversely to make the area scanned by 7TMRE accessible for OMTHE and embedded in the same 4 mm inner diameter glass tubes that were employed for 7TMRE using US gel. In addition, a total of 42 zebrafish embryos were investigated in groups of seven at 3, 25, 48, 66, 98, and 120 h post fertilization (hpf).

### 4.3 Biofilm Preparation

Biofilms of wild‐type *Bacillus subtilis* (strain NCIB3610) were formed at a solid/air interface as follows. A liquid culture of *B. subtilis* was grown by shaking at 250 rpm for 16 h at 37 °C. A 2 µL drop of the liquid culture was placed onto plates of 1.5% or 3% agar‐MSgg.^[^ ^78^ ^]^ Liquid droplets were more loosely distributed on plates containing 1.5% agar than on 3% agar (7.8 ± 0.5 mm droplet diameter compared to 6.1 ± 0.5 mm), possibly due to slightly different drying rates of the two substrate surfaces. Plates were incubated at 30 °C for 24, 48, and 72 h prior to measurement. 12 biofilms grown on substrate containing 1.5% agar and 12 biofilms grown on substrate containing 3% agar were scanned. Of each group, four biofilms at 24, 48, and 72 h were studied after inoculation. One less biofilm was examined growing on 3% agar after 48 h due to breakage during transport.

In addition to OMTHE, biofilms were also investigated by shear rheometry (Discovery HR‐2, TA Instruments, USA). The growth process was the same as described above. For each time point, three sets of 10 biofilms from the 1.5% agar growth substrate and 15 sets from the 3% agar growth substrates were removed, mixed together and spread on the rheometer's 20 mm parallel plates. Strain sweep measurements were performed at 0.01%–100% strain with an angular frequency set at 10 rad s^−1^, followed by frequency sweep measurements at 0.05% strain amplitude and an angular frequency of 0.1 to 100 rad s^−1^.

### 4.4 Optical Measurements

Optical intensity images were acquired using a commercial high‐speed digital camera (Fastcam Mini AX100 type 540K‐S, Photron, Japan) with a proprietary complementary metal‐oxide semiconductor (CMOS) sensor, providing maximum frame rates from 4 kfps at full frame (1024 × 1024 pixels) to 540 kfps at 128 × 16 pixels with a minimum exposure time of 1.04 µs. The camera was controlled using the Photron FASTCAM Viewer Software (PFV4) and was set to a resolution of 1024 by 992 pixels, enabling a frame rate of 4500 fps and an ISO of 40 000. Exposure time was adjusted to the lighting conditions of the sample. Each image acquisition was independently triggered using a frequency generator (AFG 3022B, Tektronix, USA) that also supplied signal to the actuator amplifiers. The flow of data is shown in Figure 7A, and the different setups are summarized in Figure 7B.

**Figure 8:**
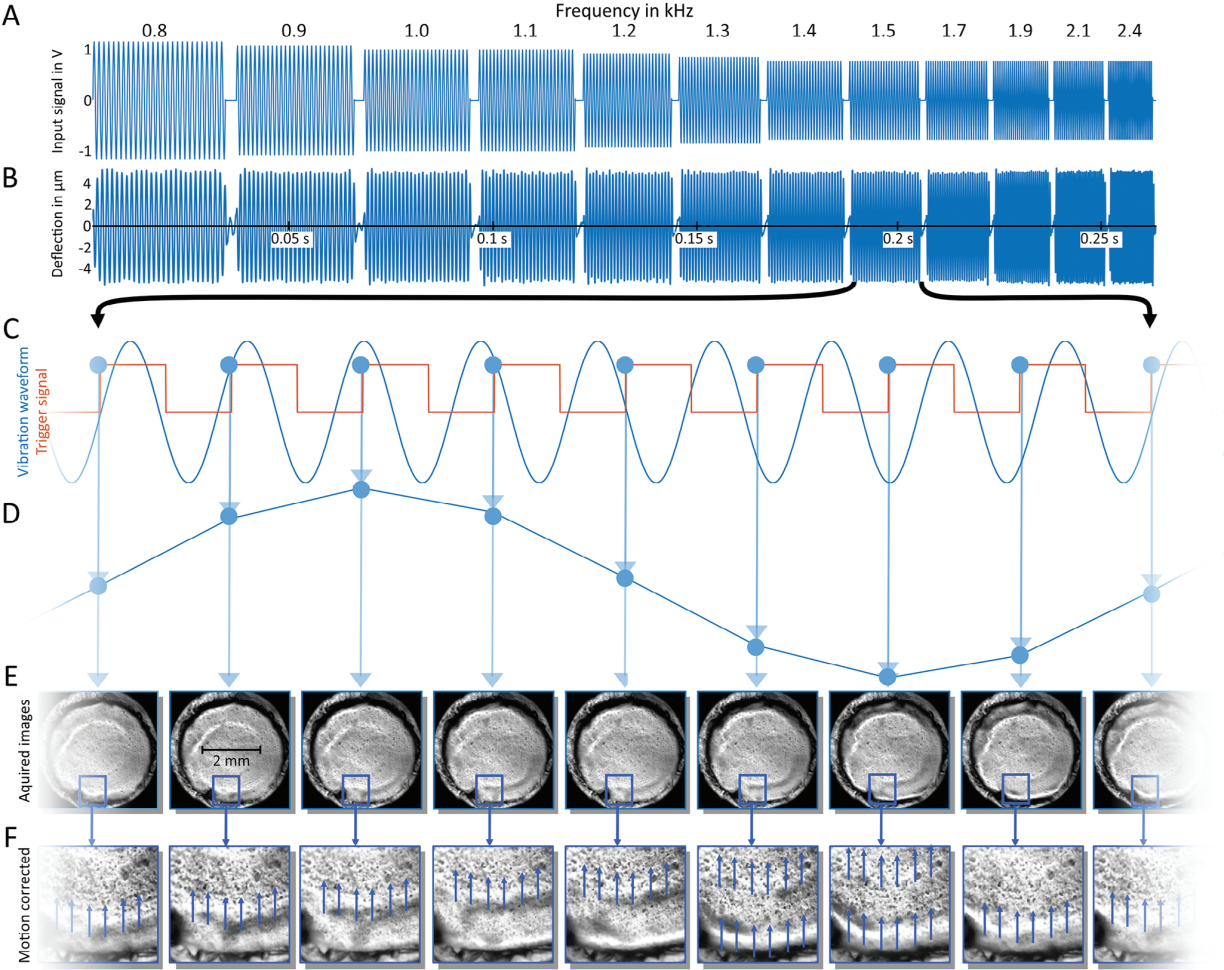
Schematic overview of image acquisition. A) Signal voltage sent to amplifier: Over a period of 0.3 s, 12 different frequencies from 800 to 2400 Hz were excited. The decrease in amplitude with increasing frequency was determined from the frequency deflection responses of the actuators. B) Experimental deflections measured by laser distance measurement at the actuator matching the input waveforms shown in A) and revealing maximum deflection amplitudes in the order of 4 µm. C) Enlarged view of the electrical signal sent to the mechanical excitation unit (blue) and the TTL trigger signal sent to the camera (orange) for one frequency. The wave is stroboscopically sampled, resulting in the reconstructed wave shown in D). E) Images acquired with the wave already visible. A magnified detail from each image is shown in F), where the propagating wavefront is clearly visible even before motion estimation (animated waves are shown in the Movie S2, Supporting Information).

As shown in the simulations and previously described, using multiple harmonic frequencies for sample excitation mitigates the effects of standing wave and wave voids in time‐harmonic elastography.^[^ ^41^, ^79^ ^]^ We therefore consecutively excited multiple different frequencies as shown in **Figure** **8**A. Eight images per vibration cycle were acquired at equidistant time increments over 3 cycles. To ensure steady–state time‐harmonic oscillations with minimized transient deflections, we acquired no images within the first 4 vibration cycles of each frequency. The limited frame rate of 4500 fps, which was below the Nyquist frequency of some of the high‐frequency oscillations, required stroboscopic sampling, as shown in Figure 8C. Here, an intended mismatch between vibration period and frame rate yielded aliased frequencies at lower spectral bins, which were precisely known due to time‐harmonic particle motion. As a result, stroboscopic sampling was not limited by the maximum frame rate of our optical system but by the minimum exposure time. While exposure time could easily be reduced by increasing sample illumination, the maximum frame rate was an intrinsic feature of the chosen high‐speed camera and expensive to increase. Notably, the natural surface texture and light reflectance of all studied systems provided enough features in the intensity images for displacement estimation. Wave propagation was visible to the naked eye when displayed in a cine loop after serial acquisition of optical intensity images, synchronized to the external actuator in a stroboscopic sampling scheme (see Movie S2, Supporting Information). Rigid image registration was used for suppression of long wavelengths related to compression waves. No other compression wave filter was applied downstream in the processing pipeline. An overview of all experimental setups used in this study is given in Table S1 (Supporting Information).

#### 4.4.1 OMTHE in Homogeneous Phantoms

All homogenous phantoms based on US gel or agar were consecutively investigated at all 12 frequencies of 0.8, 0.9, 1.0, 1.1, 1.2, 1.3, 1.4, 1.5, 1.7, 1.9, 2.1, and 2.4 kHz. Image resolutions in quadratic pixel sizes were 2 µm, 4 µm (Axio Observer transmission microscope, Carl Zeiss Jena, Germany), 2.3 µm, 4.6 µm (Axioplan reflection microscope, Carl Zeiss Jena, Germany), and 10 µm (LAOWA CA Dreamer macro lens, Venus Optics, China). The phantoms were exited perpendicular to the image plane by a piezo actuator (APA35XS, Cedrat Technologies, France), which was driven by a piezo actuator amplifier (PX200, PiezoDrive, Australia), with the lenses focused on a layer below the surface. An overview of the different setups is provided in Figure 7B. To measure the displacement induced by the piezo actuator, laser distance measurements were performed using a laser distance sensor (OD5000‐C30T05, Sick AG, Germany). The laser was directed at the front plate of the piezo, a measurement across the full frequency range was triggered and 100 000 data points were acquired with a cycle time of 12.5 µs. The detected displacement amplitude was on the order of 4 µm following the input signal without notable delay and jitter (Figure 8B).

#### 4.4.2 OMTHE in Heterogeneous Phantoms

A heterogenous agar phantom slice of 2‐cm diameter and 3 mm thickness was placed in a petri dish and vibrated in the imaging plane at all 12 frequencies given above. Optical images with a resolution of 11.6 × 11.6 µm^2^ were captured at an exposure time of 10 µs using the macro lens described for the homogeneous phantoms. The focus plane was placed in the middle of the transparent sample.

#### 4.4.3 OMTHE in Zebrafish

A glass tube containing the adult fish was mechanically excited along the body axis, through the imaging plane by the piezo actuator. The full OMTHE frequency range was examined and images with a pixel size of 8.3 × 8.3 µm^2^ were captured with an exposure time of 20 µs using the macro lens described above for homogeneous phantoms. The zebrafish embryos were embedded in US gel and placed in a 4‐mm diameter bore hole covered with microscopy glass for stabilization and wave guiding. While it is possible to mount the embryos in different positions in this setup (such as those used to image the brain), embryos were left in their natural sideways orientation in this study. The slides and embryos were excited perpendicular to the image plane at the full frequency range. Images were then acquired using the transmission microscope used in homogeneous phantoms with an image focus plane within the transparent zebrafish embryos to acquire xy‐waves with an image pixel size of 2 × 2 µm^2^. To increase the dynamic optical range, images were acquired at three different exposure times (20, 40, and 62.48 µs). To quantify regional differences, the resulting *SWS* maps were then manually segmented into yolk, notochord, myochord, and full body (excluding yolk) regions.

#### 4.4.4 OMTHE in Biofilms

Biofilms and their supporting substrate made of 1.5% or 3% agar as explained above were placed in a petri dish of 2‐cm diameter that was horizontally vibrated using the piezo actuator and macro lens described above, with an exposure time of 33 µs and a pixel size of 18 × 18 µm^2^. Both the core and the growing periphery of the biofilm were analyzed, while wrinkles growing outside the focus plane of the lens were excluded.

### 4.5 Magnetic Resonance Elastography

#### 4.5.1 Tabletop MRE

A compact 0.5‐Tesla MRE setup described previously,^[^ ^80^ ^]^ was used to provide ground truth values for the US gel and homogenous agar phantoms. Samples were investigated in glass tubes of 3.75 mm diameter and 15 cm length which were mounted on a piezoelectric driver (Piezosystem Jena, Germany). Due to the cylinder geometry, vibrations were induced exclusively along the principal cylinder axis ensuring Bessel‐like wave propagations from the cylinder walls toward the center of the samples. Vibration frequencies were identical to the ones employed in OMTHE (12 frequencies from 0.8 to 2.4 kHz). Cylindrical wave fields were encoded using an adapted spin‐echo sequence (repetition time, TR = 3 s; echo time, TE = 38 ms) equipped with MEG of 392 mT m^−1^ amplitude along the main vibration direction and a frequency synchronized with the mechanical oscillation. FoV was 9.6 × 9.6 mm^2^ with a voxel size of 0.15 × 0.15 × 3 mm^3^, leading to a total scan time of 1:33 h per sample. After unwrapping, global *SWS* was derived from Bessel fits as described in ref. [80].

#### 4.5.2 High Field MRE (7TMRE)

Adult zebrafish and heterogeneous agar phantoms were investigated in a preclinical 7‐Tesla MRI scanner (Bruker Biospec, Germany) as previously described.^[^ ^19^ ^]^ In brief, zebrafish were placed in a 4 mm inner diameter glass tube sealed by a rubber plug while the agar phantom was placed in a 16 mm wide and 22 mm deep plastic tube. Air cavities between fish and glass tube were filled with ultrasound gel to ensure sufficient wall contact for good wave transmission. The samples were mechanically vibrated mainly along the principal cylinder axis of the sample tubes using an air‐cooled piezoelectric actuator (ATA 200, Cedrat Technologies, France). Images were acquired by a 5‐mm bore volume‐resonator coil for zebrafish and a 20‐mm head coil for the phantom (Rapid Biomedical, Germany). Waves were encoded using an adapted spin‐echo sequence (TR = 3 s, TE = 38 ms) equipped with MEG of 392 mT m^−1^ amplitude along the main vibration direction and a frequency synchronized with the mechanical oscillation. Vibration frequencies in zebrafish were 1.0, 1.2 and 1.4 kHz consistent to our previous work of MRE in zebrafish.^[^ ^19^ ^]^ FoV was 4 × 4 mm^2^ with a voxel size of 60 × 60 × 600 µm^3^. Total measurement time was 48 min. In the phantoms, the vibration frequencies were adjusted to 0.8, 0.9, 1.0, 1.1, 1.2, 1.3, and 1.4 kHz to cover the OMTHE frequencies and to avoid higher frequencies with insufficient wave amplitude due to the limited power of the MRE actuator. FoV and voxel sizes were 17 × 17 mm^2^, and 0.113 × 0.113 × 1 mm^3^. Total measurement time was 2:20 h.

### 4.6 Simulations

Multifrequency complex wavefields were simulated based on a finite difference scheme with Sommerfeld boundary conditions given as Matlab (R2022a, The MathWorks, USA) code by Hirsch et al.^[^ ^35^ ^]^ and provided upon request. Field components were simulated by a horizontal or vertical planar source term for the x‐ and y‐components, respectively. Each complex wave was converted into 24 real‐valued time series over three vibration periods to which 10% white noise was added. The frequency‐ and time‐resolve wave images were taken as relative xy‐displacement functions of the coordinates of an object image with unit intensity and randomly placed features of 0.1 full width at half maximum. Maximum displacement amplitudes were scaled to 10% of the pixel size. The resulting series of encoded multifrequency harmonic displacement images were fed into the HOF and MDEV pipelines as used for the experimental data and described below. Further simulation parameters were: 500 × 500 pixels matrix size, 29.609 µm pixel edge size, 0.2 to 3.9 kHz vibration frequencies resulting in a range of 2.96 to 57.7 waves per image.

### 4.7 Image Postprocessing

All processing steps were implemented in Matlab. Zebrafish embryo images, which were captured with different exposure times, were combined prior to analysis using the Matlab function makehdr. Prior to motion estimation, all images were corrected for global (rigid) motion using the elastix toolbox (version 5.1.0)^[^ ^81^ ^]^ applied to the time series of each vibration frequency (Figure 8F). To remove light intensity changes, each image was normalized using contrast‐limited adaptive histogram equalization included in the Matlab function adapthisteq. Motion‐corrected and normalized images were then subjected to HOF‐based xy‐displacement estimation according to Equation (10) in the Appendix. Multifrequency wave inversion based on MDEV inversion was performed according to Equation (13) using the finite gradient scheme given in Equation (16) with a quadratic kernel size of 20 pixels. Total processing time for decoding xy‐displacement fields of 12 frequencies with a total of 325 images of 992 × 1024 pixels was 449 s on a quad core Intel i7‐6700 processor with 41 GB RAM. Multifrequency wave inversion was performed pixel by pixel with negligible computational cost. 7TMRE data and heterogenous phantom data were processed using wavenumber‐based MDEV, which is publicly available at https://bioqic‐apps.charite.de.^[^ ^82^ ^]^

### 4.8 Statistical Analysis

All statistical analysis was done in Matlab. To test if the myotome and notochord of zebrafish embryo were significantly different, a Wilcoxon signed rank test was used. Linear regression was used to find the relationship between zebrafish embryo length and stiffness as well as biofilm age and stiffness. When linear regression analysis was performed, Pearson's linear correlation coefficient R together with *P* values compared to the constant model was reported. Group values were reported as mean and standard deviation. If not otherwise noted, a two‐sample t‐test was used to determine if two groups were significantly different and *P* values below 0.05 were considered significant.

## 5 Appendix

### 5.1 Wave Modes Observed in OMTHE

Vibrating the sample with the actuators shown in Figure 7B induces elastic deformation on the surface and inside the bulk of the sample. Figure 1A provides an overview of the different types of waves that can arise in OMTHE. Given a homogeneous half‐space with infinite extension in the z dimension, out‐of‐plane (z) deflections are generated by Rayleigh waves, whose propagation speed is within 5% accuracy of the *SWS* of the underlying half‐space for incompressible materials.^[^ ^37^, ^61^ ^]^ Thus, in first approximation, Rayleigh waves probe the shear modulus of the subsurface material. The situation is different in plates, where z‐polarized waves, called Lamb waves, propagate with a speed depending on plate thickness and deflection symmetry.^[^ ^38^ ^]^ Because of this geometry bias, Lamb waves in thin layers (such as biofilms) should be avoided by decoding only the in‐plane shear horizontal (SH, xy‐) waves using optical flow analysis. In incompressible half‐space materials, SH surface waves are plane strain waves, which propagate with the same wave speed as the bulk SH waves in greater depths.^[^ ^38^ ^]^ Therefore, SH‐ or xy‐encoded waves are favored by OMTHE as they probe the same shear modulus parameter both at the surface and in deeper tissue layers. An unfavorable scenario in OMTHE is Love waves that may occur in layered half‐spaces when xy‐waves are encoded and the thickness of the surface layer, which must be softer than the substrate, exceeds multiple wavelengths. The speed of Love waves is a complex function of layer thickness and material properties, making Love waves difficult to analyze without additional information.^[^ ^83^ ^]^ However, the conditions neccessary for Love waves are unlikely to occur in our proposed scenarios of transparent tissue, half‐spaces or thin films but could be observed in other applications. Considering subsurface experiments on the skin, Rayleigh waves remain unaffected by Love waves due to their vertical polarization and the fact that skin *SWS* is higher than that of subcutaneous fat or muscle tissue.^[^ ^84^ ^]^ In essence, based on SH‐encoded wave modes or Rayleigh waves, OMTHE can measure *SWS* in different materials and scenarios. In the transparent zebrafish embryo, for example, focusing a microscope on a cross section through the embryo allows bulk SH wave encoding in the xy‐plane using optical flow algorithms. Similarly, whenever surfaces of opaque materials are investigated, xy‐encoding can be used to ensure that the measured *SWS* is related to the material properties of the surface layer. We therefore analyzed SH waves at surfaces in adult zebrafish and biofilms. Out‐of‐plane (z‐) waves are favorable at layer surfaces such as the skin when probing subsurface properties based on Rayleigh wave propagation under the assumption of incompressibility. The Rayleigh wave mode in OMTHE is preliminarily addressed in the supplemental note 3 by showing subsurface stiffness changes of the in vivo human biceps muscle due to voluntary muscle contraction.

### 5.2 xy‐Displacement Estimation

In‐plane, shear horizontal (xy‐) motion is recovered from the recorded sequence of optical intensity images I(r,t) based on optical flow estimation. Optical flow estimators are used to calculate motion vectors that transform one image into another.^[^ ^85^ ^]^ OMTHE assumes that the dominating motion component that is encoded in I(r,t), is the time‐harmonic deflection field given as

(1) ur,t=Reu^reitω=u^′rcostω−u^′′rsintω

ω=2πf denotes the angular drive frequency *f* with which the sample is vibrated. In 2D, the coordinate vector is r=(x,y)   while $\hat{u}$ denotes spatial oscillations with real and imaginary parts ${\hat{u}}^{\prime }$ and ${\hat{u}}^{\prime \prime }$, i.e., $\hat{u}={\hat{u}}^{\prime }+i{\hat{u}}^{\prime \prime }$. Optical flow estimates motion as $u=(u_{x},u_{y})$ between two image frames based on Taylor series approximation of image signals. A method will be introduced to estimate the complex‐valued motion field $\hat{u}=\left({\hat{u}}_{x},{\hat{u}}_{y}\right)$ at frequency *f*. To begin with, we adapt the most common solution to optical flow by Horn and Schunck, which minimizes the following objective function:^[^ ^85^ ^]^

(2) $$E\;\left(u\right)=E_{1}\;\left(u\right)+\lambda E_{2}\;\left(u\right)$$

For each t, with

(3) $$E_{1}\;\left(u\right)=\int {\left(\frac{\partial I}{\partial t}+\left(u\cdot \nabla \right)I\right)}^{2}dr\;$$

and

(4) $$E_{2}\;\left(u\right)=\int {\left|\nabla u\right|}^{2}dr$$

*E*_1_(**u**) enforces constant brightness while the regularized *E*_2_(**u**) enforces spatial smoothing. Equation (2) is minimized by solution of the Euler‐Lagrange equation:

(5) $$\frac{\partial I}{\partial t}\nabla I+\left(\nabla I\nabla I^{T}\right)u=\lambda \nabla^{2}u$$

where λ is the Lagrange multiplier. For this equation, we may consider any displacement **u**, in particular, a time‐harmonic motion as in (1). Inserting the ansatz $u\left(r,t\right)=\hat{u}\left(r\right)e^{it\omega }$ for a fixed ω, and integrating over a period *T*  =  2π/ω, we obtain.

(6) $$\frac{1}{T}\int_{-T/2}^{T/2}\frac{\partial I}{\partial t}\nabla Idt+\left(\frac{1}{T}\int_{T/2}^{T/2}\left(\nabla I\nabla I^{T}\right)dt\right)\hat{u}=\lambda \nabla^{2}\hat{u}$$

The first term on the left‐hand side can be identified as coefficients of the Fourier series while the term in the middle is the mean of ∇*I*∇*I^T^* over one period. To stabilize the solution and reduce numerical costs, we proceed with:

(7) $$m\left[\left(\nabla I\right){\left(\nabla I\right)}^{⊺}\right]\hat{u}+F\;\left[\frac{\partial I}{\partial t}\nabla I\right]=\lambda \nabla^{2}\;\hat{u}$$

where *m* denotes the mean over a time period and F is the temporal Fourier transform. The discrete Laplacian ∇^2^ on the right‐hand side can be approximated as $\nabla^{2}\hat{u}\approx \bar{\hat{u}}-\hat{u}$ such that $\bar{\hat{u}}$ denotes the weighted average in the neighborhood of $\hat{u}$ at discrete positions (*k*, *l*):^[^ ^86^ ^]^

(8) $$\bar{\hat{u}}\;\left(k,l\right)=\frac{1}{N}\sum_{n=-N}^{N}\sum_{m\neq n}\hat{u}\left(k+n,l+m\right)$$

*N* denotes the size of the Laplacian kernel. In OMTHE, *N* is scaled with pixel size *h* of an image by the empirically determined kernel size *N*  = 2^6^  µ*m*/*h*, which ensures that the smoothing capacity of $\bar{\hat{u}}\left(k,l\right)$ remains constant over a range of resolutions. Inserting Equation (8) into Equation (7) leads to:

(9) $$m\left[\left(\nabla I\right){\left(\nabla I\right)}^{⊺}\right]\hat{u}+F\;\left[\frac{\partial I}{\partial t}\nabla I\right]=\lambda \left(\bar{\hat{u}}-\hat{u}\right)$$

This can be solved iteratively for $\hat{u}$ for each drive frequency ω:

(10) $$\left(m\left[\left(\nabla I\right){\left(\nabla I\right)}^{⊺}\right]+\lambda I\right)\cdot \;\hat{u}{\left(\omega \right)}^{n+1}=\lambda {\bar{\hat{u}\left(\omega \right)}}^{n}-F\left[\frac{\partial I}{\partial t}\nabla I\right]$$

where I is the identity matrix. In each iteration step *n*, $\hat{u}\left(\omega \right)$ is filtered by a median filter with the same kernel size *N* as used for the Laplacian kernel in Equation (8).^[^ ^87^ ^]^

### 5.3 Multifrequency Shear Wave Inversion

Following the assumption that elastic tissue properties dominate *SWS*, i.e., *G*′ ≫ *G*″, where G′ and G″ denote storage and loss modulus, respectively, of the complex shear modulus G* = *G*′ + i*G*″, *SWS* can be obtained from the magnitude modulus:

(11) $$SWS=\sqrt{\frac{\left|G^{*}\right|}{\rho }}$$

Material density ρ is assumed to be 1kg/*L*, as suggested in the MRE guidelines.^[^ ^36^ ^]^ |G*| is obtained from direct inversion of motion field $\hat{u}\left(\omega \right)$ using the Helmholtz equation, which is in magnitude representation:^[^ ^35^ ^]^

(12) $$\left|G^{*}\right|\;\left|\nabla^{2}\hat{u}\left(\omega \right)\right|=\omega^{2}\;\left|\hat{u}\left(\omega \right)\right|$$

Single frequency inversion schemes of the lossless Helmholtz equation suffer from a number of problems such as inhomogeneous illumination of the tissue due to standing wave nodes. Under ideal noise‐free conditions, it would be possible to recover correct moduli even for nodes with zero displacement because Helmholtz inversion relies on spatial derivatives, i.e., analysis of the curvature of the wave. However, when noise is present, this numerical analysis is highly unstable, requiring averaging over multiple field components and *M* excitation frequencies ω_*m*_ to stabilize the estimation of *SWS*:^[^ ^41^ ^]^

(13) $$\left|G^{*}\right|=\rho \frac{\sum_{j=1}^{2}\sum_{m=1}^{M}\omega_{l}^{2}\left|{\hat{u}}_{j}\left(\omega_{m}\right)\right|}{\sum_{j=1}^{2}\sum_{m=1}^{M}\left|\nabla^{2}{\hat{u}}_{j}\left(\omega_{m}\right)\right|}$$

It is important to note that OMTHE, unlike MRE, does not apply bandpass filters to $\hat{u}\left(\omega_{m}\right)$ for suppression of compression waves and noise prior to inversion. Compression waves are cancelled out by rigid image registration prior to motion estimation, as shown in Figure 1C, while OMTHE SNR is on the order of 51 to 58 dB based on the Donoho method,^[^ ^42^, ^62^ ^]^ which is ≈2000 to 4000 times higher than MRE SNR. Instead of smoothing, OMTHE suppresses unwanted signal by higher dimensional finite gradient schemes, as proposed by Anderssen and Hegland:^[^ ^88^ ^]^

(14) $$a_{k,l}:=y_{k+s_{1},l}\;-y_{k-s_{1},l}$$

(15) $$b_{k,l}:=y_{k,l+s_{2}}\;-y_{k,l-s_{2}}$$

(16) $$\begin{array}{cc} {\left(\nabla {\hat{u}}_{j}\right)}_{k,l} & =\frac{1}{2\left(2r_{1}+1\right)\left(2r_{2}+1\right)}\\ & \;\times \left(\frac{1}{s_{1}h}\sum_{m=-r_{1}}^{r_{1}}\sum_{n=-r_{2}}^{r_{2}}a_{k+n,l+m}+\frac{1}{s_{2}h}\sum_{m=-r_{1}}^{r_{1}}\sum_{n=-r_{2}}^{r_{2}}b_{k+n,l+m}\right) \end{array}$$

*y*_*k*,l_ are the pixels in the wave image at positions (*k*, *l*), *h* denotes pixel size, and *r*_1_, *r*_2_, *s*_1_, *s*_2_ define the range of the neighborhood in the two image directions. In this work, *s*_1_ =   *s*_2_ = 10 was used.

## Conflict of Interest

The authors declare no conflict of interest.

## Supporting information



Supporting Information

Supplemental Movie 1

Supplemental Movie 2

## Data Availability

The data that support the findings of this study are available from the corresponding author upon reasonable request.
