# Supplementary material for: Rapid Stiffness Mapping in Soft Biologic Tissues With Micrometer Resolution Using Optical Multifrequency Time‐Harmonic Elastography
Source: Adv Sci (Weinh). 2024 Dec 16;12(8):2410473. doi: 10.1002/advs.202410473 (PMC11848577; doi:10.1002/advs.202410473)
Supplement: Supplementary file 1 — Supporting Information [file ADVS-12-2410473-s002.docx]

# Rapid stiffness mapping in soft biologic tissues with micrometer resolution using multifrequency time-harmonic elastography

Jakob Jordan^1^, Noah Jaitner^1^, Tom Meyer^1^, Luca Brahmè^2,3^, Mnar Ghrayeb^4,5^, Julia Köppke^2,3^, Oliver Böhm^1^, Stefan Klemmer Chandia^1^, Vasily Zaburdaev^6,7^, Liraz Chai^4,5^, Heiko Tzschätzsch^8^, Joaquin Mura^9^, Anja I.H. Hagemann^2,3^, Jürgen Braun^8^, Ingolf Sack^1^

1 Department of Radiology, Charité – Universitätsmedizin Berlin​
2 Department of Hematology/Oncology, Charité – Universitätsmedizin 10117 Berlin
3 German Cancer Consortium (DKTK)—German Cancer Research Center (DKFZ), 69120 Heidelberg
4 The Center for Nanoscience and Nanotechnology, The Hebrew University of Jerusalem, Edmond J. Safra Campus, Jerusalem, 91901, Israel
5 Institute of Chemistry, The Hebrew University of Jerusalem, Edmond J. Safra Campus, Jerusalem, 91901, Israel
6 Department of Biology, Friedrich-Alexander-Universität Erlangen-Nürnberg, 91058 Erlangen
7 Max-Planck-Zentrum für Physik und Medizin, 91054 Erlangen, Germany
8 Institute of Medical Informatics, Charité – Universitätsmedizin Berlin
9 Department of Mechanical Engineering, Universidad Técnica Federico Santa María, Santiago, Chile.

Contents

[Supplementary materials: Optical stiffness mapping in soft biomaterials by multifrequency time-harmonic elastography](#_Toc152874202)

[Supplementary note 1: Optical elastography methods 2](#_Toc152874203)

[Supplementary Figure 1: Visualizing wave images in zebrafish and biofilm 3](#_Toc152874204)

[Supplementary Figure 2: Development of zebrafish embryo anatomy 4](#_Toc152874205)

[Supplementary table 1: Overview of experiments with relevant parameters used in this study 5](#_Toc152874206)

[Supplementary note 2: On spatial resolution in biphasic fluid-solid biofilms 6](#_Toc152874207)

[Supplementary note 3: OTHE in anisotropic muscle tissue 7](#_Toc152874208)

[Supplementary note 4: Rheology measurement of biofilms 8](#_Toc152874209)

[References 8](#_Toc152874210)

## Supplementary note 1: Optical elastography methods

Optical methods have a long history in elastography [1]. The first optical approach for detecting compressive deformation at a micrometer scale was optical coherence elastography (OCE). This phase-sensitive method enables the detection of nanometer-scale axial displacement between two line-scans [2, 3]. With frame rates of up to 1 MHz, OCE has an impressive acquisition speed [4]. However, when strain results from static compression, the conversion of displacement into a stiffness map becomes complex [5]. Consequently, OCE has been used to track wave fronts propagated by transient acoustic impulses, a key principle in ultrasound elastography [2]. A significant advantage of transient waves is that shear wave speed can be directly estimated from acoustic phase velocity. Recently, transient waves at surfaces detected by ultrafast optical cameras have been used to explore subsurface properties across a broad range of resolutions, from single cells [6, 7] to large phantoms [8, 9]. These studies indicate that optical elastography based on high-speed cameras might be more versatile than OCE, even though it cannot provide 3D displacement cubes of subsurface structures. A strength of optical cameras is their high fidelity in capturing in-plane (xy-) motion at high spatial resolutions. This allows optical flow detection algorithms [10] to retrieve the xy-components of the deformation field. xy-deformation (strain) waves probe intrinsic tissue properties by imposing a plane-strain scenario. However, due to periodic changes in optical reflectance, cameras can also record surface wave which are polarized out-of-plane (see supplementary chapter *Supplementary note 3: In vivo muscle*). Such z-polarized surface waves, called Rayleigh waves, bear a strong resemblance to shear waves in the bulk of the material beneath the surface [11]. For incompressible materials, the difference in $SWS$ between Rayleigh and bulk shear waves is only about 5%, opening a window into the assessment of subsurface properties of, for example, skeletal muscle through the skin [12]. Moreover, capturing shear waves polarized in-plane allow stiffness mapping with the high spatial resolution provided by the optical system. However, due to $SWS$ dispersion, stiffness is an intrinsic tissue parameter only when the dynamic stimulation frequency is known. This condition is readily fulfilled by continuously exciting time-harmonic motion and establishing a continuous flux of shear wave energy as a remote probe of tissue stiffness. Parker et al. used reverberant OCE based on single-frequency harmonic wave fields in transparent corneal tissue [13] and underneath the dura mater of the mouse brain [14] while Flé et al. detected continuous vibrations with a high-speed camera in live mouse oocytes [7]. However, these methods are single-frequency and cannot be scaled, limiting their spatial resolution for multiscale stiffness mapping.

## Supplementary Figure 1: Visualizing wave images in zebrafish and biofilm


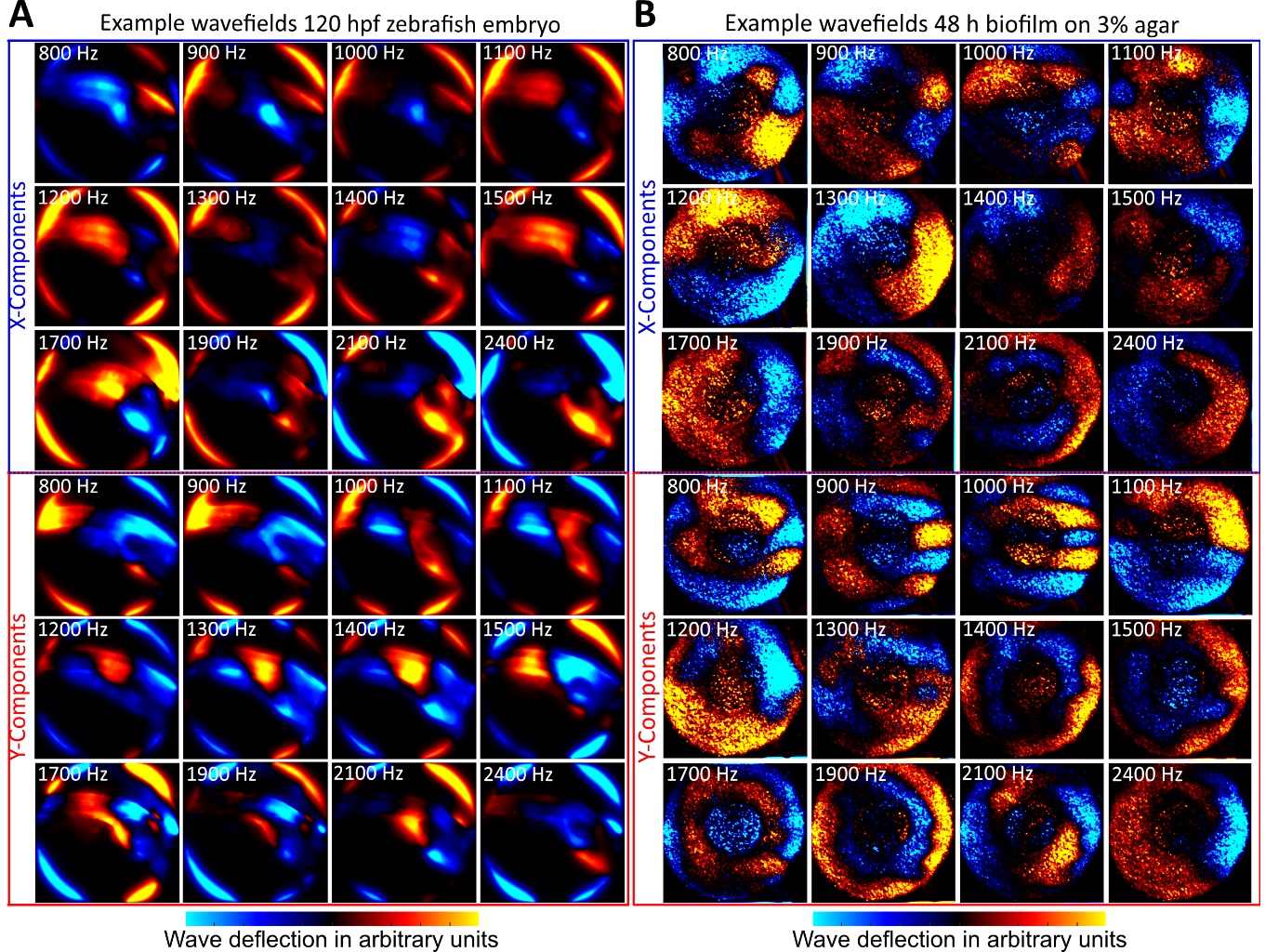


**Figure S1** Representative wave images across all measured frequencies and directions for **(A)** one embryo at 120 hours post fertilization (hpf) and **(B)** one biofilm at 48 h of growth on a substrate containing 3% agar.

## Supplementary Figure 2: Development of zebrafish embryo anatomy


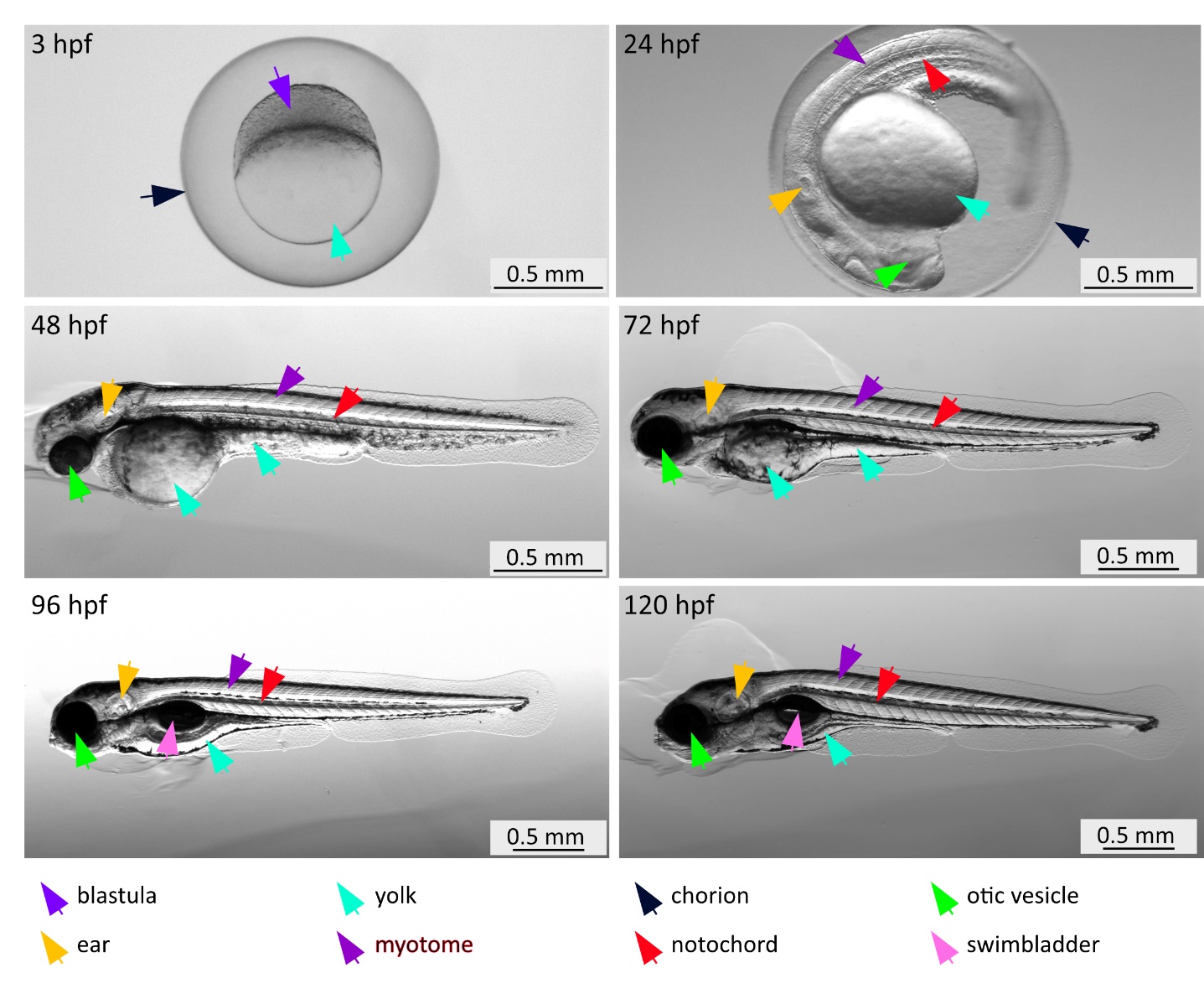


**Figure S2** Development of zebrafish embryos at between 3 hpf to 120 hpf with labeled organs.

## Table S1: Overview of experiments with relevant parameters used in this study

| Material, tissue | Optical system | Camera pixel Resolution (µm) | Exposure time (µs) | Frequencies (Hz) | Number of time steps | Number of repetitions | Laplacian filter size (pixel) | Anderssen stencil  width (pixel) | Excitation direction |
| --- | --- | --- | --- | --- | --- | --- | --- | --- | --- |
| Phantom | Axio Observer  (Carl Zeiss Jena) | 2 | 20 | 800  900  1000  1100  1200  1300  1400  1500  1700  1900  2100  2400 | 8 | 3 | 31 | 21 | horizontal |
|  | Axioplan  (Carl Zeiss Jena) | 2.3 | 20 |  | 8 | 3 | 27 | 21 | horizontal |
|  | Axio Observer  (Carl Zeiss Jena) | 4 | 40 |  | 8 | 3 | 15 | 21 | horizontal |
|  | Axioplan  (Carl Zeiss Jena) | 4.6 | 40 |  | 8 | 3 | 13 | 21 | horizontal |
|  | LAOWA CA Dreamer  (Venus Optics, China) | 10 | 40 |  | 8 | 3 | 7 | 21 | horizontal |
| Adult Zebrafish | LAOWA CA Dreamer | 8.3 | 20 |  | 8 | 3 | 7 | 21 | vertical |
| Zebrafish Embryo | Axio Observer  (Carl Zeiss Jena) | 2 | 20, 40,  62.48 |  | 8 | 3 | 31 | 21 | horizontal |
| Biofilm | LAOWA CA Dreamer  (Venus Optics, China) | 18 | 33 |  | 8 | 3 | 3 | 21 | horizontal |
| Muscle | LAOWA CA Dreamer  (Venus Optics, China) | 25 | 100 | 275 375 550 750 1000 | 8 | 8 | % | 21 | vertical |

## Supplementary note 2: On spatial resolutions of OMTHE in biphasic fluid-solid biofilms

The wrinkled surface geometry formed by biofilms is of great interest not only from a biological perspective but also from a mechanical point of view. As shown in figure 6B and Supplementary figure S3, the core regions exhibited abundant wrinkles of approximately 210 ± 40 µm in cross-section. In contrast, fewer but larger wrinkles of approximately 540 ± 50 µm width were visible in the periphery. These vessel-like structures in the core and periphery contain water that can move freely, as opposed to confined water compartments within cells or intercellular spaces [15]. Unconfined water within pores, wrinkles, or sulci acts as a lubricant that facilitates displacement of tissue at interfaces through shear forces [16]. As a result, a material with many slip interfaces behaves softer than the same material with welded (dry) interfaces. In shear wave elastography, slip interfaces pose a challenge because they resemble cracks in the material and cause discontinuities in the wave phase. Correct estimation of material properties near slip interfaces requires precise knowledge of the boundary conditions, which is practically infeasible. Therefore, MRE often compiles wave discontinuities into soft properties at or near the location of slip boundaries, where leaps in the phase gradient occur. Such steep wave gradients are indistinguishable from short wave numbers, which in turn encode soft tissue properties. If the crack is thin, these values are clearly incorrect. However, from a coarse-grained perspective, these values encode a boundary layer of the thickness of the point spread function of the inversion method [17]. Such a boundary layer reflects a mixture of the shear resistance of the interface and the shear stiffness of the adjacent solid compartments. Thus, stiffness at coarser resolutions reflects the sum of the microelements, including their cross-links and cracks, all of which contribute to the behavior of the material at the length scale of the measurement. Our observation of soft properties in the biofilm core appears to reflect the presence of abundant slip interfaces due to water-filled microchannels as seen in the micrographs presented in figure S3. Reducing water content in this region would likely affect stiffness toward higher values as the lubricating effect of water decreases. Similarly, wrinkles in the periphery can be viewed as bulging and wobbly structures that are easily displaced by shear forces [17]. As a result, OTHE detects soft values in these regions. Notably, detected stiffness properties do not appear to be affected by optical properties, as suggested by the bright core area in the biofilms shown in figure S3, while dark intensities are seen in the stiffness maps.


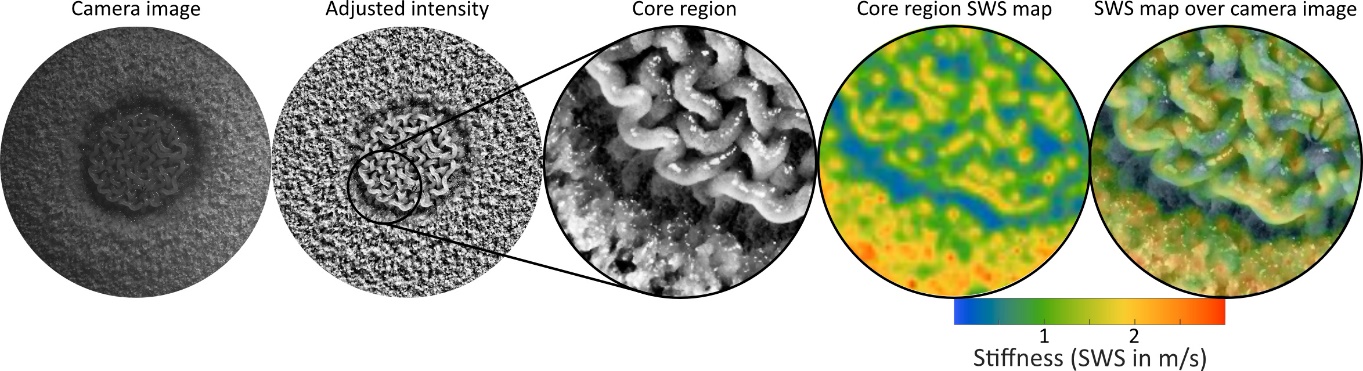


**Figure S3** From left to right: acquired raw image, intensity-adjusted image, enlarged core region, and $SWS$ map of enlarged core region. A match between stiffness and slip interfaces caused by wrinkles is visible in the $SWS$ map.

## Supplementary note 3: In vivo muscle

The ability of skeletal muscles to change stiffness and to generate mechanical force enables the body to move. As such, muscle stiffness is intimately linked to muscle function. However, quantitative measurement of changes in muscle function due to pathology or injury remains challenging [18]. As a possible option to overcome these challenges, we tested the ability of OMTHE to probe subsurface stiffness properties in vivo. This test mode was based on Rayleigh surface waves (z-deflection component), which have wavelengths in a similar range as the bulk shear waves in the underlying muscle [12]. OMTHE in muscle was designed to excite shear waves perpendicular to the main fiber direction. Given a transverse isotropic symmetry of the biceps brachii muscle, the propagation direction of the shear waves would also be transverse to the fibers, as shown in figure S4A. This scenario corresponds to a slow transverse wave propagation, in which shear waves probe perpendicular shear modulus $\mu_{12}$ in contrast to fast transverse waves, which are related to parallel shear modulus $\mu_{13}$ [19]. A sketch of the experimental setup can be seen in figure S4B.

#### OMTHE detection of z-surface waves

Unlike xy-waves, z-deflections are 3D phenomena requiring stereo cameras, optical coherence techniques, or laser vibrometers when detected by light [9]. Alternatively, one can analysis relative intensity changes due to light scattering. Light is diffracted when surfaces are displaced along the surface normal (z), resulting in apparent motion patterns corresponding to the intensity changes recorded by the optical system. Thus, after temporal Fourier transform of $I(x,y,t)$, we obtain

$\hat{I}\left( x,y,\omega\right)\approx{a_{0}e}^{i(\mathbf{kr+}t\omega)}\hat{=}\hat{u}_{z}\left( x,y, \omega\right)$. (S1)

$a_{0}$ denotes the relative deflection amplitude along $z$ since the true physical deflection, $u_{0z}$ , is obscured by image intensities. However, wave inversion is based on normalized ratios of $\hat{\mathbf{u}}\boldsymbol{(}\omega\boldsymbol{)}$, making the absolute wave amplitude irrelevant.


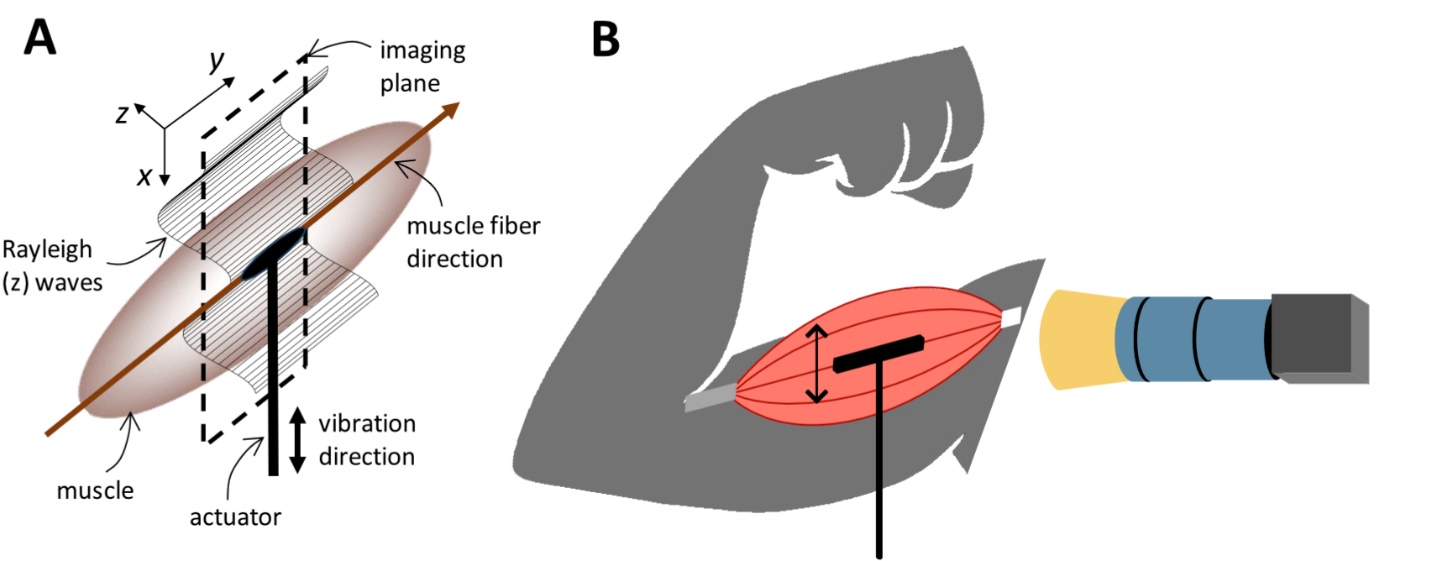


**Figure S4** *Experimental setup for in vivo OMTHE measurement in biceps brachii muscle* **(A)** *Close-up view of biceps brachii muscle. The actuator vibrates in the x direction, perpendicular to the muscle fiber direction. The induced Rayleigh wave travels in the x direction and deflects in the z direction.* **(B)** *Setup of the muscle experiment. Biceps brachii muscle shown in red. Movement of actuator indicated by black arrow.*

#### OMTHE methods in skeletal muscle

The biceps brachii muscles of the right arm of 11 healthy volunteers (8 male, 3 female; age: 30 ± 9 years) were investigated after written informed consent was obtained. The study was approved by the ethical committee of the Charité - Universitätsmedizin Berlin (EA4/040/22). The actuator setup of OMTHE in muscle was designed to induce the slow-transverse component of the shear waves in a sagittal plane. Rayleigh wave motions were captured at the skin surface in a lateral position using a field of view (FOV) of 2.11 $\times$ 2.05 cm^2^, a resolution of 25 $\times$ 25 µm^2^, and an exposure time of 100 µs. Waves were excited outside the FOV using a voice coil (AR-50, Monacor, Germany) driven by a commercial audio amplifier (LD-Systems PA 1600-X, Adam Hall Group, Germany) perpendicular to the fiber direction (figure S4A). Frequencies were excited at 275 Hz, 375 Hz, 550 Hz, 750 Hz, and 1000 Hz, providing similar wavelengths relative to the size of the FOV as in the other experiments conducted in this study. Harmonic z-waves were extracted from the optical images according to equation S1 and analyzed by wave profile fitting as shown in figure S5. Two experiments were performed in each subject, once in a relaxed state of the muscle and once during maximum voluntary contraction.


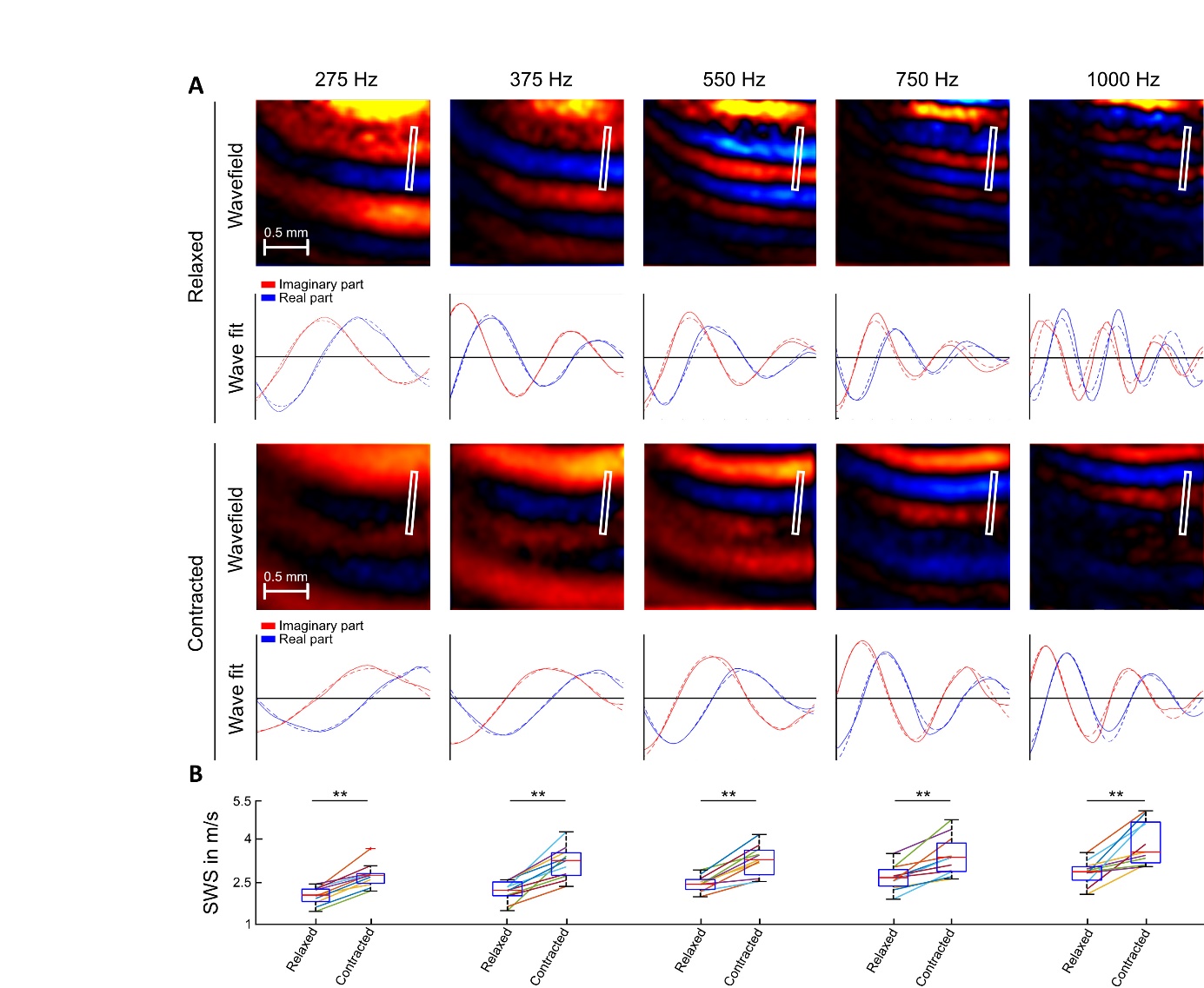


**Figure S5** In-vivo OMTHE in biceps brachii muscle. **(A)** Out-of-plane wavefield components across all frequencies measured for relaxed and contracted biceps brachii. A line profile of the real and imaginary parts of the waves is shown below the wavefield, with the wave fit represented by dotted lines. **(B)** Percent change in stiffness between relaxed and contracted muscle for all subjects and frequencies.

#### OMTHE results in skeletal muscle

Figure S5A shows the Rayleigh wavefields in a volunteer for each frequency for the relaxed and contracted muscle. For each frequency, the imaginary and real parts of the waves are plotted across the profile along with wave fits to determine the wavelengths and estimate stiffness. Across all frequencies, $SWS$ values were higher in the contracted than in the relaxed muscles (P = 8.6 × 10^-3^) with no frequency-dependent effect size (P = 0.39, figure S5B). Mean values across frequencies showed a 38% increase in stiffness with contraction (contracted: 3.4 ± 0.8 m/s, relaxed: 2.5 ± 0.5 m/s, P = 3.9 × 10^-4^).

#### Discussion on preliminary OMTHE in skeletal muscle

We chose skeletal muscle to demonstrate this sensitivity based on Rayleigh waves because muscle function is directly related to changes in stiffness, and measuring muscle stiffness in vivo remains a challenge for many current elastography methods [20-22] due to pronounced shear wave damping, anisotropy, and load-dependent stiffness variation [23]. Our preliminary study in biceps muscle was designed to provide an outlook into potential diagnostic applications. Long scan times in MRE make clinical stiffness scans at defined loading states inefficient for clinical use in this area. While ultrasound-based muscle elastography has a role in the clinic, comprehensive stiffness function tests based on combined EMG-elastography examinations have not yet been established [24]. Surface-based wave detection methods such as OMTHE could provide solutions to current challenges in diagnostic muscle imaging. Although further validation with respect to anisotropy is needed, our OMTHE results fall into the range reported in the literature for the perpendicular component of relaxed muscle stiffness. For example, MRE in human biceps brachii muscle showed $SWS$ of 2.3 ± 0.9 m/s for the perpendicular stiffness component and 5.4 ± 2.4 m/s for the parallel stiffness components at drive frequencies from 75 Hz to 118 Hz [19]. Ultrasound elastography, without consideration of anisotropy, found values ranging from 1.7 to 5.1 m/s in an unspecified range of frequencies, and identified sex, age, and elbow position as influencing factors [25].

# References

1. Kennedy, B.F., P. Wijesinghe, and D.D. Sampson, *The emergence of optical elastography in biomedicine.* Nature Photonics, 2017. **11**(4): p. 215-221.

2. Nahas, A., et al., *From supersonic shear wave imaging to full-field optical coherence shear wave elastography.* Journal of biomedical optics, 2013. **18**(12): p. 121514-121514.

3. Li, Y., et al., *Ultrahigh-sensitive optical coherence elastography.* Light: Science & Applications, 2020. **9**(1): p. 58.

4. Liu, C.-H., et al., *Ultra-fast line-field low coherence holographic elastography using spatial phase shifting.* Biomedical Optics Express, 2017. **8**(2): p. 993-1004.

5. Schmitt, J.M., *OCT elastography: imaging microscopic deformation and strain of tissue.* Optics express, 1998. **3**(6): p. 199-211.

6. Grasland-Mongrain, P., et al., *Ultrafast imaging of cell elasticity with optical microelastography.* Proceedings of the National Academy of Sciences, 2018. **115**(5): p. 861-866.

7. Fle, G., et al., *Imaging the subcellular viscoelastic properties of mouse oocytes.* Proc Natl Acad Sci U S A, 2023. **120**(21): p. e2213836120.

8. Torres, J., et al., *Optical micro-elastography with magnetic excitation for high frequency rheological characterization of soft media.* Ultrasonics, 2023. **132**: p. 107021.

9. Zorgani, A., et al., *Optical elastography: tracking surface waves with digital image correlation.* Phys Med Biol, 2019. **64**(5): p. 055007.

10. Horn, B.K.P. and B.G. Schunck, *Determining Optical-Flow.* Proceedings of the Society of Photo-Optical Instrumentation Engineers, 1981. **281**: p. 319-331.

11. Royston, T.J., H.A. Mansy, and R.H. Sandler, *Excitation and propagation of surface waves on a viscoelastic half-space with application to medical diagnosis.* Journal of the Acoustical Society of America, 1999. **106**(6): p. 3678-3686.

12. Zhang, X. and J.F. Greenleaf, *Estimation of tissue's elasticity with surface wave speed.* J Acoust Soc Am, 2007. **122**(5): p. 2522-5.

13. Zvietcovich, F., et al., *Reverberant 3D optical coherence elastography maps the elasticity of individual corneal layers.* Nature Communications, 2019. **10**.

14. Gary, R.G., et al., *Fluid compartments influence elastography of the aging mouse brain.* Physics in Medicine & Biology, 2023. **68**(9): p. 095004.

15. Wilking, J.N., et al., *Liquid transport facilitated by channels in Bacillus subtilis biofilms.* Proceedings of the National Academy of Sciences, 2013. **110**(3): p. 848-852.

16. Papazoglou, S., et al., *Scatter-based magnetic resonance elastography.* Phys Med Biol, 2009. **54**(7): p. 2229-41.

17. Papazoglou, S., et al., *Horizontal shear wave scattering from a nonwelded interface observed by magnetic resonance elastography.* Phys Med Biol, 2007. **52**(3): p. 675-84.

18. Bilston, L.E. and K. Tan, *Measurement of Passive Skeletal Muscle Mechanical Properties In Vivo: Recent Progress, Clinical Applications, and Remaining Challenges.* Ann Biomed Eng, 2014: p. [Epub ahead of print] DOI: 10.1007/s10439-014-1186-2.

19. Papazoglou, S., et al., *Shear-wave group-velocity inversion in MR elastography of human skeletal muscle.* Magn Reson Med, 2006. **56**(3): p. 489-497.

20. Ringleb, S.I., et al., *Applications of magnetic resonance elastography to healthy and pathologic skeletal muscle.* J Magn Reson Imaging, 2007. **25**(2): p. 301-9.

21. Ashir, A., et al., *Skeletal Muscle Assessment Using Quantitative Ultrasound: A Narrative Review.* Sensors, 2023. **23**(10): p. 4763.

22. Ličen, U. and Ž. Kozinc, *Using Shear-Wave Elastography to Assess Exercise-Induced Muscle Damage: A Review.* Sensors, 2022. **22**(19): p. 7574.

23. Bilston, L.E. and K. Tan, *Measurement of passive skeletal muscle mechanical properties in vivo: recent progress, clinical applications, and remaining challenges.* Annals of biomedical engineering, 2015. **43**: p. 261-273.

24. Yang, Y., et al., *Explorative study using ultrasound time-harmonic elastography for stiffness-based quantification of skeletal muscle function.* Z Med Phys, 2024.

25. Eby, S.F., et al., *Shear wave elastography of passive skeletal muscle stiffness: influences of sex and age throughout adulthood.* Clinical biomechanics, 2015. **30**(1): p. 22-27.
