# Supplementary figures and images for: Rapid Stiffness Mapping in Soft Biologic Tissues With Micrometer Resolution Using Optical Multifrequency Time‐Harmonic Elastography
Source: Adv Sci (Weinh). 2024 Dec 16;12(8):2410473. doi: 10.1002/advs.202410473 (PMC11848577; doi:10.1002/advs.202410473)

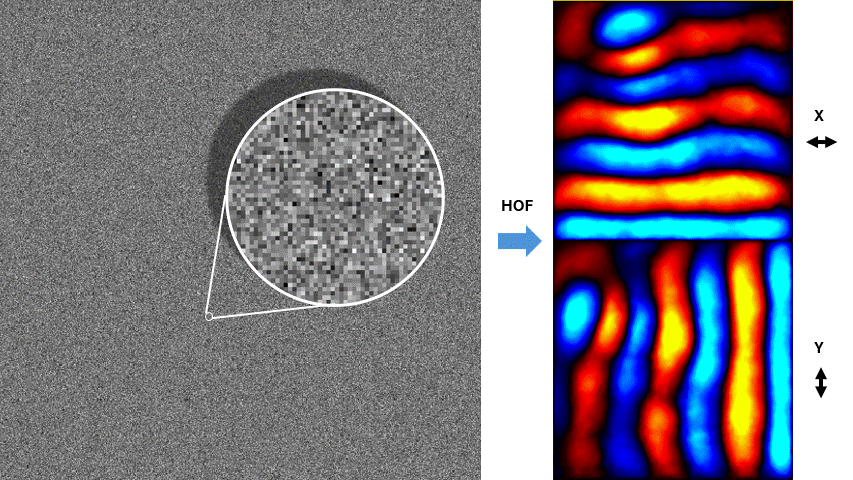

Supplement: Supplementary file 2 — Supplemental Movie 1 [file ADVS-12-2410473-s003.gif]
